# Sex-specific associations between brominated flame retardants exposure and phenotypic age acceleration in NHANES 2005–2010

**DOI:** 10.3389/fpubh.2025.1513757

**Published:** 2025-04-08

**Authors:** Weiliang Kong, Yilian Xie, Yina Jin

**Affiliations:** ^1^Department of Respiratory and Critical Care Medicine, Key Laboratory of Respiratory Disease of Ningbo, First Affiliated Hospital of Ningbo University, Ningbo, China; ^2^Department of Hepatology, First Affiliated Hospital of Ningbo University, Ningbo, China

**Keywords:** phenotypic age, aging, brominated flame retardants, NHANES, sex-specific

## Abstract

**Background:**

Exposure to brominated flame retardants (BFRs) has been linked to age-related diseases. This study investigates the associations between both individual and combined BFRs exposures and phenotypic age acceleration (PhenoAgeAccel) in U.S. adults.

**Methods:**

Data from 3,908 U.S. adults from NHANES 2005–2010 were analyzed. Generalized linear regression models (GLMs) assessed the associations between individual BFRs and PhenoAgeAccel, while weighted quantile sum (WQS) regression and Bayesian Kernel Machine Regression (BKMR) analyses were used to evaluate the effects of combined BFRs exposures.

**Results:**

GLMs indicated significant positive associations between several BFRs and PhenoAgeAccel, including PBDE28 (β = 0.55, 95% CI: 0.13, 0.96), PBDE85 (β = 0.42, 95% CI: 0.11, 0.74), PBDE47 (β = 0.39, 95% CI: 0.04, 0.75), PBDE99 (β = 0.38, 95% CI: 0.07, 0.68), and PBDE154 (β = 0.37, 95% CI: 0.04, 0.70). RCS analysis confirmed nonlinear dose–response relationships for PBDE47 and PBDE99 (P for nonlinearity = 0.03361 and 0.0233, respectively). Stratified analyses revealed that males were more susceptible to BFRs exposure effects, particularly for PBDE99 (P for interaction = 0.027) and PBDE209 (P for interaction = 0.005). The WQS regression showed a significant association between combined BFRs exposure and increased PhenoAgeAccel (β = 0.504, 95% CI: 0.071, 0.937), with PBB153 and PBDE153 as key contributors. BKMR analysis indicated a trend of increasing PhenoAgeAccel with higher BFR exposure levels, primarily driven by PBDE99.

**Conclusion:**

This study highlights the significant positive associations between individual and combined BFR exposures and PhenoAgeAccel, with males potentially being more vulnerable to these effects.

## 1 Introduction

Brominated flame retardants (BFRs) are a group of chemicals widely used in plastics, electronics, and furniture to enhance flame resistance. However, due to their persistence in the environment and potential for bioaccumulation, BFRs have emerged as environmental pollutants of global concern. These substances are prevalent in air, soil, water, and biological samples such as human serum and breast milk (1). Despite the phase-out of certain BFRs in many countries (2), they remain in the environment and continue to expose humans through various pathways, including inhalation of dust, dietary intake, and dermal contact (3). Some studies have indicated that BFRs exhibit thyroid toxicity, neurotoxicity, and reproductive and developmental toxicity, posing significant health risks (4–6). Compounds such as polybrominated diphenyl ethers (PBDEs) and polybrominated biphenyls (PBBs), due to their lipophilicity and long half-lives, can accumulate in the human body over time. This means that despite their gradual discontinuation, human exposure to BFRs persists, presenting ongoing risks (7).

Aging is commonly defined as the gradual decline in physiological functions and systemic integrity that occurs over time, leading to increased vulnerability to diseases and an elevated risk of death (8). It involves the progressive accumulation of damage at the cellular, molecular, and tissue levels, resulting in the deterioration of bodily systems and a reduced ability to maintain homeostasis (8). Aging is a multifaceted process influenced by genetic, environmental, and lifestyle factors, manifesting in various biological, functional, and cognitive changes that contribute to the overall aging phenotype. To provide a more precise measure of an individual’s aging state, taking into account their overall health, various biomarkers of aging have been developed (9), such as phenotypic age (10), biological age (11), DNA methylation (12), and HD (13). Among these, phenotypic age—a composite biomarker based on biological markers and mortality risk—has demonstrated high accuracy in predicting disease incidence and mortality (14). While, phenotypic age acceleration (PhenoAgeAccel) is derived from phenotypic age and serves as an indicator of whether an individual’s biological aging process is faster or slower than expected, given their chronological age. A positive PhenoAgeAccel value reflects accelerated aging, meaning the individual’s biological state is older than their chronological age, while a negative value indicates decelerated aging, suggesting a younger biological state. This metric has been linked to increased susceptibility to age-related diseases, functional decline, and mortality. As such, PhenoAgeAccel provides a valuable framework for understanding how external factors, including environmental exposures, may influence the aging process. Given the dual challenges of environmental pollution and population aging, it is critical to investigate how environmental substances affect the aging process. Previous studies have suggested that BFRs exposure are linked to several age-related diseases, including cancer (15), cardiovascular diseases (16), neurodegenerative conditions (17), and metabolic disorders (18). Additionally, *in vitro* studies have shown that BFRs can induce apoptosis (19), which is one of the 12 hallmarks of biological aging (14).

Therefore, this study aims to explore the potential association between BFRs exposure and phenotypic age acceleration (PhenoAgeAccel) using data from the National Health and Nutrition Examination Survey (NHANES). By examining this relationship, the study seeks to provide new evidence on the role of environmental exposure in the aging process, contributing to the growing body of research on the impact of environmental pollutants on human health.

## 2 Materials and methods

### 2.1 Design

This study analyzed data from the 2005–2010 NHANES cycles, spanning three consecutive survey periods. Although BFRs data is available through 2016, a methodology changes in C-reactive protein (CRP) measurement occurred in the 2011–2012 cycle with the introduction of high-sensitivity CRP. Since CRP is a critical marker for calculating phenotypic age, data after 2010 were excluded to ensure methodological consistency.

**Figure 1:**
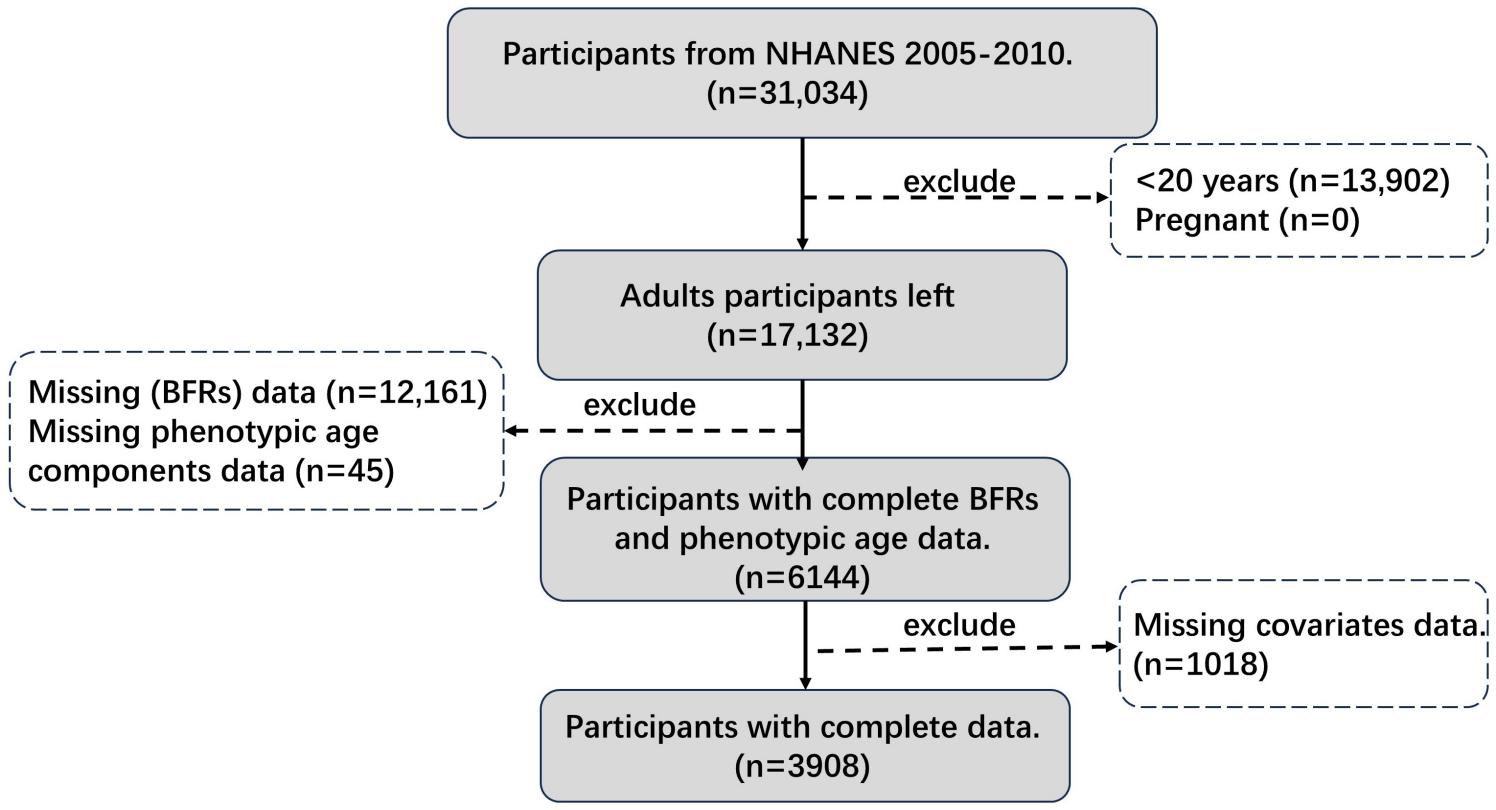
Participants flow chart.

NHANES, conducted by the CDC, is a nationally representative survey employing a complex, multi-stage sampling design to evaluate the health and nutrition of the U.S. population. Participants undergo interviews, physical exams, and biospecimen collection at mobile examination centers. For this analysis, individuals aged 20 and older were included, providing information on demographics, socio-economic status, dietary intake, chronic diseases, and BFRs exposure from the 2005–2010 cycles. After applying specific inclusion and exclusion criteria, a total of 3,908 participants were selected for the final analysis, as outlined in Figure 1.

### 2.2 BFRs exposure

BFRs were measured in serum using automated liquid–liquid extraction followed by gas chromatography coupled with high-resolution mass spectrometry. In line with previous studies, serum BFR concentrations were used to reflect individual exposure levels. The analysis included polybrominated biphenyl (PBB153) and eight polybrominated diphenyl ethers (PBDEs) with detection rates above 65% (20). The selected PBDEs were: 2,4,4′-Tribromodiphenyl ether (PBDE28), 2,2′,4,4´-Tetrabromodiphenyl ether (PBDE47), 2,2′,3,4,4´-Tetrabromodiphenyl ether (PBDE85), 2,2′,4,4′,5-Pentabromodiphenyl ether (PBDE99), 2,2′,4,4′,6-Pentabromodiphenyl ether (PBDE100), 2,2′,4,4′,5,5´-Hexabromodiphenyl ether (PBDE153), 2,2′,4,4′,5,6´-Hexabromodiphenyl ether (PBDE154), and Decabromodiphenyl ether (PBDE209). In this study, values below the limit of detection (LOD) were imputed using limit divided by the square root of two, in accordance with the NHANES analytical guidelines (21). This approach is widely adopted in environmental health research. Moreover, previous studies have assessed the sensitivity of various imputation methods for handling values below the detection limit, providing support for LOD/√2 as a reasonable and widely accepted estimation method (22).

### 2.3 Phenotypic age acceleration

Aging acceleration was assessed using phenotypic age, which is calculated based on various biomarkers and a specific algorithm. To control for chronological age, PhenoAgeAccel was determined as the residual from a regression of phenotypic age on chronological age. Participants were categorized into two groups: those with accelerated aging (PhenoAgeAccel ≥ 0) and those with delayed aging (PhenoAgeAccel < 0).

Phenotypic age was calculated using the following formula (14):


$$\mathrm{Phenotypic}\;\mathrm{Age}=141.50+\frac{\ln\left[-0.00553\times \ln\left(1-\mathrm{MortalityScore}\right)\right]}{0.09165}$$

Where:


$$\mathrm{MortalityScore}=1-\exp\left(\frac{-1.51714\times \exp\left(xb\right)}{0.0076927}\right)$$

And:

𝑥𝑏 = −19.907–0.0336 × Albumin+0.0095 × Creatinine+0.1953 × Glucose+0.0954 × ln(CRP) − 0.0120 × Lymphocyte Percentage+0.0268 × Mean Cell Volume+0.3306 × Erythrocyte Distribution Width + 0.00188 × Alkaline Phosphatase+0.0554 × Leukocyte Count+0.0804 × Chronological Age. Here, “𝑥𝑏 “represents the linear combination of biomarkers used in the model.

### 2.4 Covariation

Based on previous literature, covariates that have been proven to be associated with environmental factors and biological aging were included in the analysis. These covariates were: age, sex, race/ethnicity, poverty-to-income ratio (PIR), BMI, marital status, education level, smoking status, alcohol consumption, healthy dietary status (assessed using the Healthy Eating Index-2015 [HEI-2015] (23)), physical activity level, and the presence of chronic diseases, including hypertension, diabetes mellitus (DM), cardiovascular disease (CVD), and cancer, Additionally, the corresponding survey year was included. Hypertension and DM were determined through both self-reports and clinical measurements, while CVD and cancer were self-reported. Estimated glomerular filtration rate (eGFR) was calculated using the Chronic Kidney Disease Epidemiology Collaboration (CKD-EPI) equation. Participants were classified by PIR as low (<1.3), middle (1.3–3.5), and high (>3.5); BMI was categorized based on WHO classifications (normal, overweight, obesity); alcohol consumption was divided into non-drinkers, moderate drinkers (1–3 drinks/day), and heavy drinkers (≥4 drinks/day); and physical activity level was categorized as active, inactive, moderate, or others (24).

### 2.5 Statistical methods

Although NHANES employs a complex sampling design, weighting was not applied in this study due to the limitations in incorporating weights into mixture analyses. For baseline characteristics, continuous variables were presented as means with standard deviations (SD), while categorical variables were expressed as counts and percentages. Differences in continuous variables between the delayed and accelerated aging groups were assessed using Student’s *t*-tests, and chi-square tests were employed for categorical variables.

Generalized Linear Models (GLMs) were used to evaluate the associations between BFRs(log-transformed) and PhenoAgeAccel. Three models were applied: the crude model, which was unadjusted; Model 1 adjusted for age, sex, and race/ethnicity; and Model 2 further adjusted for BMI, PIR, marital status, education, physical activity, smoking, alcohol consumption, hypertension, diabetes, cardiovascular disease, cancer, HEI-2015, and survey year. Restricted cubic spline (RCS) analysis was conducted to investigate the dose–response relationship between BFRs exposure and PhenoAgeAccel after adjusting for all confounders. Subgroup and interaction analyses were also performed based on age and sex.

In the second phase, quantile sum (WQS) regression was used to assess the effect of BFRs mixture exposure on PhenoAgeAccel. The bootstrap method (10,000 iterations) was applied to assign weights to each contaminant, identifying the most influential components in the mixture. Contaminant weights ranged from 0 to 1. This method not only captures real-world co-exposures more effectively but also enhances sensitivity in identifying key predictors compared to univariate analysis. The data were randomly split into 40% training and 60% testing samples, with 10,000 bootstrap iterations.

Additionally, Bayesian Kernel Machine Regression (BKMR) was employed to visualize the joint exposure-response relationship between BFRs mixtures and biological aging risk. BKMR’s strength lies in its flexibility to model exposure-response relationships, accounting for potential nonlinear and non-additive effects often seen in environmental epidemiology. The exposure-response functions were modeled using a Gaussian process, and the analysis was run with 10,000 iterations of the Markov Chain Monte Carlo (MCMC) algorithm. Conditional posterior inclusion probabilities (conPIPs) were calculated to identify the key BFRs components most associated with biological aging risk. All statistical analyses were conducted by R version 4.4.12.

## 3 Results

### 3.1 Baseline characteristics of study participants

A total of 3,908 U.S. adults were included in the study and categorized into two groups based on PhenoAgeAccel: the delayed aging group (*n* = 3,004) and the accelerated aging group (*n* = 904). Participants in the accelerated aging group were significantly older, with a mean age of 54.78 ± 17.46 years, compared to 41.30 ± 17.85 years in the delayed aging group. The accelerated aging group also had a higher proportion of non-Hispanic Black individuals, lower PIR, lower HEI-2015 scores, reduced eGFR, lower education levels, and a lower rate of active physical activity. In contrast, this group exhibited higher BMI, a greater prevalence of widowed/divorced/separated individuals, and an increased incidence of smoking, hypertension, cancer, and diabetes. Furthermore, the accelerated aging group was exposed to higher levels of PBDE28, PBDE47, PBDE85, PBDE99, PBDE100, and PBDE154. Detailed information is provided in Table 1. Besides, the characteristics of study participants by sex in adults are presented in Supplementary Table S1, showing that BFRs exposure levels were significantly higher in males compared to females. Additionally, the characteristics of study participants with accelerated phenotypic age by sex are provided in Supplementary Table S2, where certain BFRs exhibited significantly higher exposure levels in males than in females.

**Table 1:** Characteristics of the study participants among U.S adults (NHANES 2005–2010).

Variable	Total (*n* = 3,908)	Delayed (*n* = 3,004)	Accelerated (*n* = 904)	*p* value
Phenotypic age	46.22 ± 20.66	41.30 ± 17.85	62.58 ± 20.96	<0.001
PhenoAgeAccel	−3.23 ± 8.40	−6.55 ± 3.72	7.80 ± 10.04	<0.001
Age	49.45 ± 17.66	47.85 ± 17.40	54.78 ± 17.46	<0.001
Age group				<0.001
<40	1,310 (33.52)	1,099 (36.58)	211 (23.34)	
40–59	1,305 (33.39)	1,027 (34.19)	278 (30.75)	
≥60	1,293 (33.09)	878 (29.23)	415 (45.91)	
Sex				0.29
Male	1,987 (50.84)	1,513 (50.37)	474 (52.43)	
Female	1,921 (49.16)	1,491 (49.63)	430 (47.57)	
Ethnicity				<0.001
Non-Hispanic White	1,971 (50.44)	1,569 (52.23)	402 (44.47)	
Non-Hispanic Black	737 (18.86)	492 (16.38)	245 (27.10)	
Mexican American	709 (18.14)	544 (18.11)	165 (18.25)	
Others	491 (12.56)	399 (13.28)	92 (10.18)	
PIR				<0.001
Low	1,096 (28.05)	763 (25.40)	333 (36.84)	
Middle	1,553 (39.74)	1,187 (39.51)	366 (40.49)	
High	1,259 (32.22)	1,054 (35.09)	205 (22.68)	
BMI				<0.001
Obesity	1,438 (36.80)	941 (31.32)	497 (54.98)	
Normal	1,102 (28.20)	941 (31.32)	161 (17.81)	
Overweight	1,368 (35.01)	1,122 (37.35)	246 (27.21)	
Marital status				<0.001
Never married	613 (15.69)	498 (16.58)	115 (12.72)	
Widowed/divorced/separated	885 (22.65)	601 (20.01)	284 (31.42)	
Married/living with partner	2,410 (61.67)	1,905 (63.42)	505 (55.86)	
Education				<0.001
Middle school or lower	453 (11.59)	317 (10.55)	136 (15.04)	
High school	1,537 (39.33)	1,139 (37.92)	398 (44.03)	
College or more	1,918 (49.08)	1,548 (51.53)	370 (40.93)	
Physical activity				<0.001
Inactive	910 (23.29)	712 (23.70)	198 (21.90)	
Moderate	480 (12.28)	391 (13.02)	89 (9.85)	
Active	1,520 (38.89)	1,224 (40.75)	296 (32.74)	
Others	998 (25.54)	677 (22.54)	321 (35.51)	
Smoke				<0.001
Never	2,040 (52.20)	1,648 (54.86)	392 (43.36)	
Former	1,033 (26.43)	766 (25.50)	267 (29.54)	
Now	835 (21.37)	590 (19.64)	245 (27.10)	
Drinks				<0.001
Former	720 (18.42)	479 (15.95)	241 (26.66)	
Mild	1,257 (32.16)	1,010 (33.62)	247 (27.32)	
Never	507 (12.97)	376 (12.52)	131 (14.49)	
Moderate	561 (14.36)	458 (15.25)	103 (11.39)	
Heavy	863 (22.08)	681 (22.67)	182 (20.13)	
Hypertension	1,590 (40.69)	1,060 (35.29)	530 (58.63)	<0.001
DM	675 (17.27)	293 (9.75)	382 (42.26)	<0.001
CVD	286 (7.32)	156 (5.19)	130 (14.38)	<0.001
Cancer	369 (9.44)	247 (8.22)	122 (13.50)	<0.001
HEI-2015	50.62 ± 13.49	50.98 ± 13.55	49.41 ± 13.21	<0.01
eGFR	93.14 ± 23.31	96.19 ± 20.70	83.02 ± 28.16	<0.001
PBB153^*^	37.99 ± 69.69	37.31 ± 70.54	40.26 ± 66.76	0.25
PBDE28^*^	11.20 ± 7.50	10.94 ± 7.38	12.08 ± 7.85	<0.001
PBDE47^*^	219.52 ± 204.75	212.58 ± 195.92	242.58 ± 230.29	<0.001
PBDE85^*^	4.94 ± 5.96	4.74 ± 5.63	5.63 ± 6.92	<0.001
PBDE99^*^	49.70 ± 61.06	47.29 ± 56.87	57.72 ± 72.73	<0.001
PBDE100^*^	45.24 ± 44.26	44.20 ± 42.82	48.70 ± 48.60	0.01
PBDE153^*^	76.61 ± 67.71	77.63 ± 69.31	73.20 ± 62.03	0.07
PBDE154^*^	4.51 ± 4.86	4.36 ± 4.62	5.01 ± 5.55	<0.01
PBDE209^*^	22.99 ± 39.75	22.74 ± 38.07	23.85 ± 44.90	0.50

Continuous variable was presented as mean ± SD and categorical variables were presented as numbers (percentages). Variables between groups were compared by Student’s *t*-tests and chi-square tests.

*Unit: pg/g.

### 3.2 Associations between individual BFRs exposures and PhenoAgeAccel

Table 2 presents the results of the GLMs, revealing significant positive associations between several BFRs and PhenoAgeAccel. Specifically, PBDE28 (β = 0.55, 95% CI: 0.13, 0.96), PBDE85 (β = 0.42, 95% CI: 0.11, 0.74), PBDE47 (β = 0.39, 95% CI: 0.04, 0.75), PBDE99 (β = 0.38, 95% CI: 0.07, 0.68), and PBDE154 (β = 0.37, 95% CI: 0.04, 0.70) were all significantly associated with PhenoAgeAccel.

**Table 2:** Associations between individual brominated flame retardants exposures and PhenoAgeAccel.

Characters	Crude model		Model 1		Model 2	
	95%CI	*P* value	95%CI	*P* value	95%CI	*P* value
PBB153	0.85 (0.60, 1.11)	<0.001	0.21 (−0.11, 0.53)	0.19	−0.03 (−0.31, 0.25)	0.83
PBDE28	1.32 (0.87, 1.77)	<0.002	0.82 (0.35, 1.29)	<0.001	0.55 (0.13, 0.96)	0.01
PBDE85	1.17 (0.82, 1.52)	<0.004	0.7 (0.34, 1.05)	<0.001	0.42 (0.11, 0.74)	0.01
PBDE47	1.23 (0.83, 1.63)	<0.003	0.71 (0.31, 1.11)	<0.001	0.39 (0.04, 0.75)	0.03
PBDE99	1.12 (0.78, 1.47)	<0.005	0.65 (0.30, 1.00)	<0.001	0.38 (0.07, 0.68)	0.02
PBDE100	1 (0.62, 1.38)	<0.006	0.58 (0.20, 0.96)	0.003	0.31 (−0.02, 0.64)	0.07
PBDE154	1.11 (0.74, 1.48)	<0.007	0.59 (0.22, 0.97)	0.002	0.37 (0.04, 0.70)	0.03
PBDE209	0.29 (−0.25, 0.83)	0.29	0.13 (−0.40, 0.67)	0.63	0.06 (−0.40, 0.52)	0.79
PBDE153	0.04 (−0.33, 0.41)	0.83	0.15 (−0.23, 0.53)	0.44	0.26 (−0.07, 0.58)	0.12

Crude model: adjusted for none. Model 1: adjusted for age, sex, ethnicity. Model 2: adjusted for age, sex, ethnicity, PIR, BMI, marital status, education, physical activity, smoke, drinks, hypertension, DM, CVD, cancer, HEI-2015, eGFR, and year.

**Figure 2:**
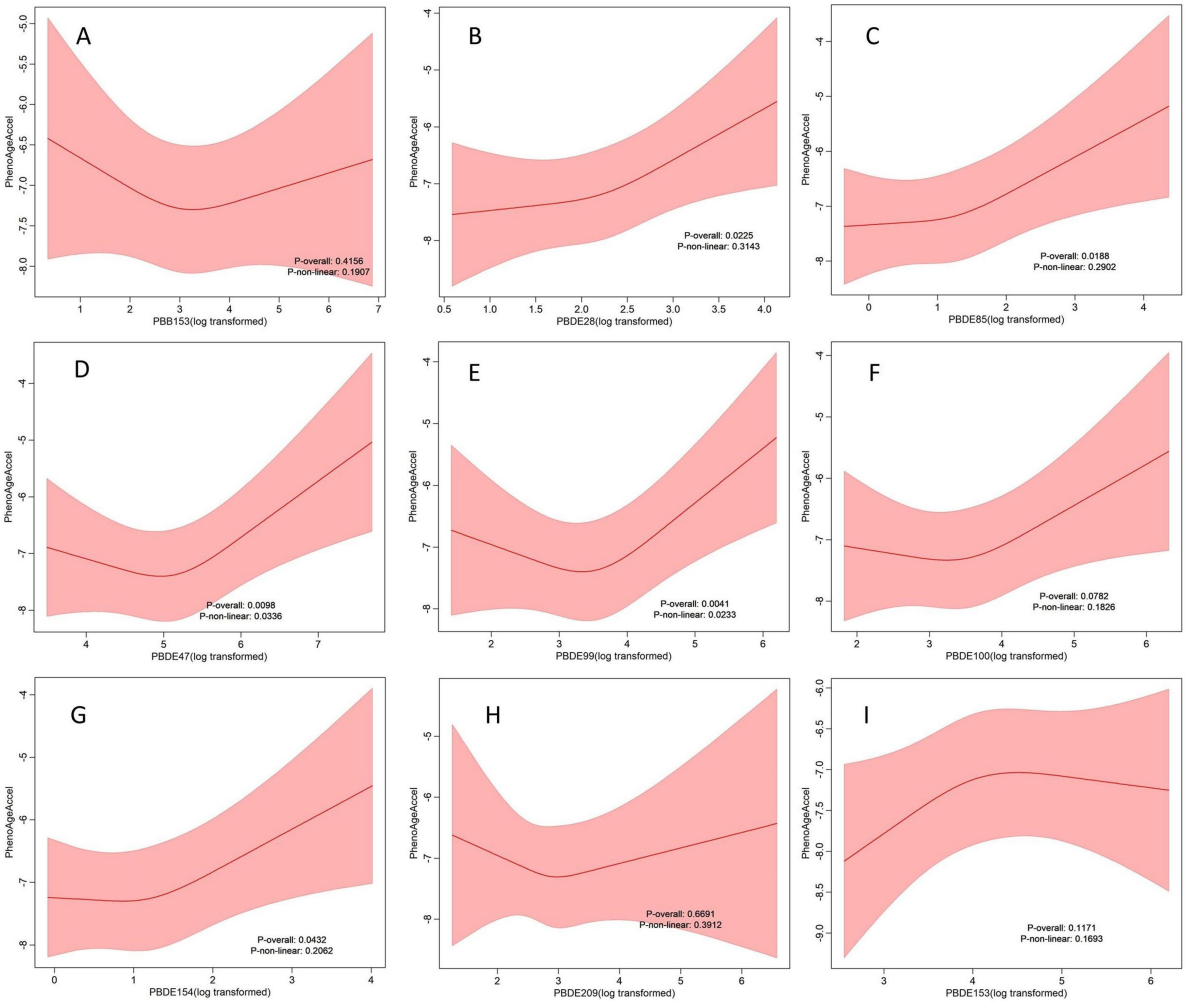
Figures **(A–I)** depict dose–response relationship between individual BFRs and PhenoAgeAccel in the sample of 3,908 U.S adults from NHANES 2005 to 2010. Red solid lines and Red dotted line represent RCS models and 95%CI, respectively. Multivariable linear regression model is used to estimate the fully adjusted coefficient in PhenoAgeAccel and corresponding 95% CI. Models were adjusted by age, sex, ethnicity, BMI, PIR, marital status, education, physical activity, smoke, drinks, hypertension, DM, CVD, cancer, eGFR, HEI-2015, and year.

RCS analysis confirmed both linear and nonlinear dose–response relationships for several PBDEs (Figure 2). Specifically, PBDE47 (P for overall = 0.0098, P for nonlinearity = 0.0336) and PBDE99 (P for overall = 0.0041, P for nonlinearity = 0.0233) exhibited significant nonlinear associations with PhenoAgeAccel, indicating that increases in exposure were associated with sharper increases in PhenoAgeAccel at higher exposure levels. In contrast, PBDE28 (P for overall = 0.0225, P for nonlinearity = 0.3143), PBDE85 (P for overall = 0.0118, P for nonlinearity = 0.2902), and PBDE154 (P for overall = 0.0432, P for nonlinearity = 0.2062) demonstrated significant overall associations without evidence of nonlinearity, suggesting that their relationships with PhenoAgeAccel were more likely linear.

The stratified analysis by age and sex revealed differential associations between BFRs exposure and PhenoAgeAccel (Supplementary Table S3). Males were more susceptible to the effects of PBB153, PBDE28, PBDE85, PBDE47, PBDE99, and PBDE154 compared to females, with significant sex interactions (P for interaction <0.0001, 0.002, 0.003, 0.017, 0.003, and 0.025, respectively). Additionally, PBDE99 showed a stronger effect in participants aged 60 and older (P for interaction = 0.027), while PBDE209 had a more pronounced effect in the 40–59 age group (P for interaction = 0.005).

### 3.3 Associations between BFRs mixture and PhenoAgeAccel

**Figure 3:**
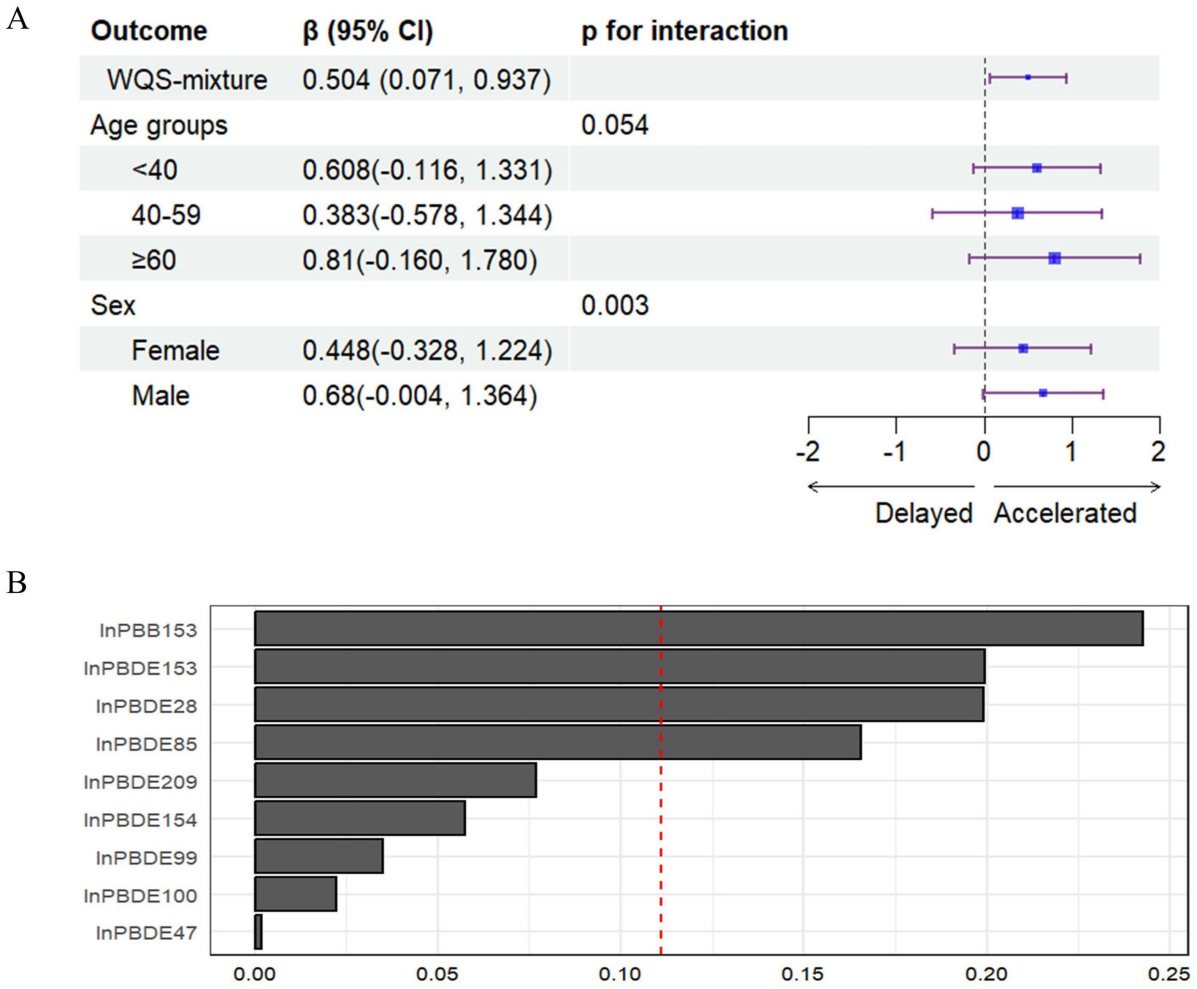
**(A)** The association between WQS mixture of BFRs and PhenoAgeAccel and stratified results by age group and sex. **(B)** Weights from weighted quantile sum regression for the mixture and PhenoAgeAccel.

When constraining the analysis in the positive direction, the WQS regression analysis (Figure 3) revealed a significant association between combined exposure to BFRs and an increase in PhenoAgeAccel (β = 0.504, 95% CI: 0.071, 0.937), with this effect being particularly pronounced among males. The chemicals that contributed most to this increase in PhenoAgeAccel were PBB153, PBDE153, PBDE28, and PBDE85. Interaction analyses indicated that males were more strongly affected by this exposure. Conversely, in the negative direction, no significant association was found (β = 0.424, 95% CI: −0.084, 0.932).

**Figure 4:**
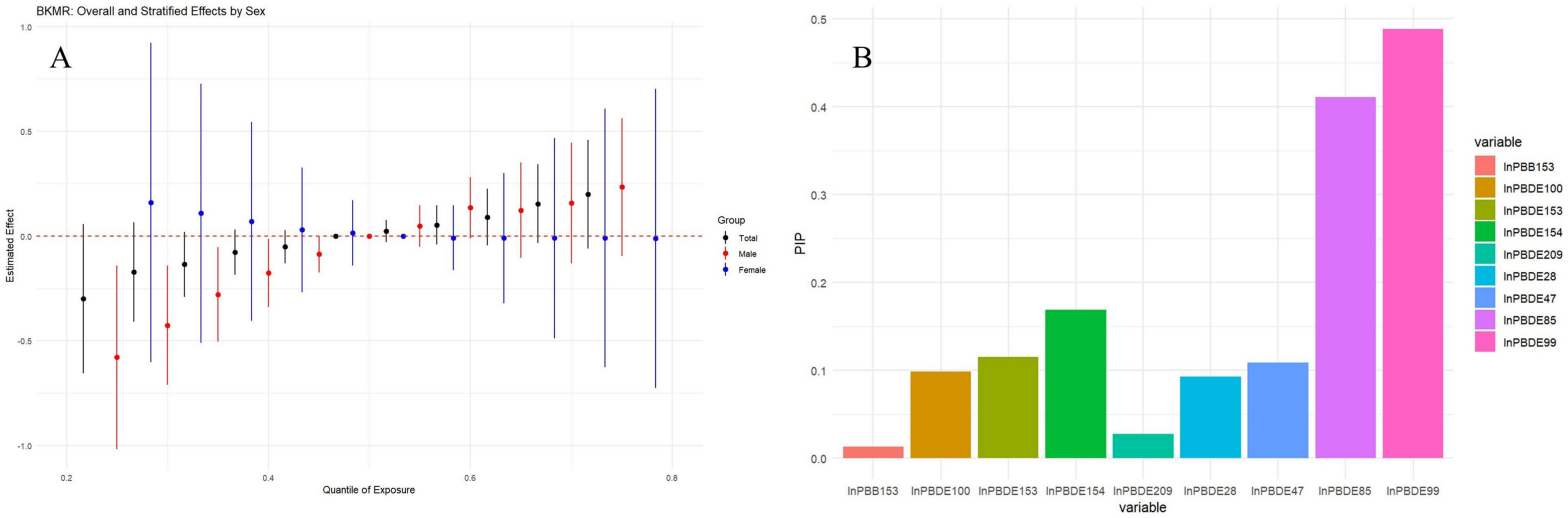
Association between combined BFRs exposure and PhenoAgeAccel analyzed by BKMR model. **(A)** Overall effects of BFRs mixture on PhenoAgeAccel and stratified results by sex at all concentrations ranged from the first quantile (10%) to the third quantile (90%) relative to the median (50%) level. **(B)** Posterior inclusion probabilities (PIPs) of each BFRs for PhenoAgeAccel. Models adjusted for age, sex, ethnicity, BMI, PIR, marital status, education, physical activity, smoke, drinks, hypertension, DM, CVD, cancer, eGFR, HEI-2015, and year.

The BKMR analysis (Figure 4A) indicated as exposure to BFRs increases from lower to higher quantiles, there is a noticeable trend in the estimated effects, suggesting that higher mixed BFRs exposure may be associated with increased PhenoAgeAccel, particularly in males (red circles). The posterior inclusion probabilities (PIPs) identified PBDE99 as the primary drivers of this effect, Other BFRs, such as PBDE28 and PBDE85, also show significant probabilities, but to a lesser extent than lnPBDE99 (Figure 4B). Males exhibited a stronger response to the BFRs mixture compared to females. Supplementary Figure S2A shows the univariate exposure-response analysis for each BFR, indicating a positive monotonic relationship between PBDE100, PBDE85, and PBDE99 with PhenoAgeAccel. In contrast, PBDE28, PBDE47, and PBDE154 exhibited a negative monotonic trend. As shown in Supplementary Figure S2B, when other chemicals were held constant at the 25th, 50th, and 75th percentiles, a positive association between PBDE100, PBDE85, and PBDE99 and PhenoAgeAccel was observed. Additionally, no statistically significant interactions between BFR components were detected (Supplementary Figure S2C).

## 4 Discussion

This study is the first to systematically examine the relationship between brominated flame retardants (BFRs) exposure and PhenoAgeAccel using U.S. NHANES data. Our findings reveal significant positive associations between several BFRs including PBDE28, PBDE85, PBDE47, PBDE99, and PBDE154 and PhenoAgeAccel. Notably, PBDE47 and PBDE99 demonstrated non-linear dose–response relationships. Both WQS mixture analysis and BKMR confirmed a positive link between BFR mixtures and PhenoAgeAccel. WQS regression identified PBB153, PBDE153, PBDE28, and PBDE85 as the primary contributors to this association, while BKMR analysis highlighted PBDE99, PBDE85, and PBDE154 as key drivers of PhenoAgeAccel. Additionally, stratified analyses revealed sex-specific differences, with males showing greater susceptibility to the adverse effects of BFR exposure.

Epidemiological studies exploring the relationship between BFRs exposure and aging remain limited. However, previous research has identified associations between BFRs and several age-related diseases. For instance, PBDE100 and PBDE153 have been independently linked to hypertension prevalence, while BFR mixtures have been associated with an increased risk of hypertension (25). Che et al. found a relationship between BFR exposure and metabolic syndrome, identifying PBB153, PBDE209, and PBDE28 as significant contributors (18). Furthermore, BFRs exposure has been positively correlated with CVD risk, with PBB153 identified as a key factor, particularly in relation to congestive heart failure and coronary heart disease (16). These findings are consistent with our study. Additionally, our study also revealed that sex modulates the relationship between BFRs exposure and PhenoAgeAccel, with males being more sensitive to PBB153, PBDE28, PBDE85, PBDE47, PBDE99, and PBDE154. One potential explanation for this sex difference lies in the influence of sex hormones, particularly testosterone and estrogen, which play critical roles in regulating metabolic and physiological processes (26). As endocrine disruptors, BFRs may interfere with these hormonal pathways, potentially leading to more pronounced metabolic dysregulation in males (27). Moreover, the higher proportion of lean body mass in males compared to females could alter the distribution, storage, and metabolism of lipophilic compounds like BFRs, further contributing to these differences (28, 29). Another plausible factor is differential exposure pathways. For example, occupational or lifestyle variations may result in higher BFRs exposure in males (30), as suggested by the data presented in Supplementary Tables S1, S2, although this warrants further investigation. Additionally, sex-based metabolic differences, such as variations in liver enzyme activity or fat metabolism, may influence the biotransformation and clearance of BFRs, exacerbating the observed disparities in sensitivity (26, 31). While these findings underscore important sex-specific variations, further studies are needed to elucidate the underlying mechanisms and to explore additional factors that may contribute to the differential susceptibility observed in males.

Although the precise mechanisms through which BFRs influence aging remain unclear, several hypotheses, supported by various studies, provide potential explanations. One mechanism involves the endocrine-disrupting properties of BFRs, particularly PBDEs. PBDEs are recognized as endocrine disruptors that can potentially interfere with estrogen receptor and thyroid hormone signaling, both of which are involved in regulating adipose tissue metabolism and maintaining metabolic homeostasis (32, 33). These disruptions are hypothesized to contribute to metabolic dysregulation, which has been associated with markers of accelerated aging in prior studies. Another mechanism that may influence aging is oxidative stress and inflammation (34). *In vitro* studies have demonstrated that exposure to PBDE-47, PBDE-209, and PBDE-99 induces oxidative stress and inflammatory responses in bronchial epithelial cells, which are linked to DNA damage and apoptosis. These processes are fundamental to cellular aging (34). Experiments involving HepG2 cells, wild-type N2 worms, and adipocytes have shown that PBDE-47 exposure may contribute to a dose-dependent increase in oxidative stress (35–37). Reactive oxygen species, a hallmark of oxidative stress, are recognized as one of the 12 hallmarks of aging. In human umbilical vein endothelial cells, PBDE-209 has been shown to induce ROS production, further linking BFR exposure to cellular senescence (38). Furthermore, epidemiological evidence suggests an association between BFR exposure and oxidative stress biomarkers, which may provide insight into potential pathways linking BFRs to biological aging. A study analyzing NHANES data from 2007 to 2016 revealed a positive association between BFR levels and oxidative stress biomarkers in U.S. adults (39). Aging presents the many characteristics including high bioavailability of oxidative stress and inflammation (40). Overall, BFRs may influence biological processes potentially relevant to aging, including endocrine disruption, oxidative stress, inflammation, and organ-specific toxicity. While these mechanisms are plausible, further research is needed to clarify their roles and interactions in relation to biological aging.

Although our study provides the first preliminary evidence linking BFRs exposure to PhenoAgeAccel, further research is necessary to validate these findings due to several limitations. First, this study’s cross-sectional design precludes establishing causal relationships between BFRs exposure and PhenoAgeAccel. The mechanisms discussed are based on associations observed in this study and prior experimental findings, and should be interpreted as hypotheses requiring further validation through longitudinal and experimental research. Second, there is currently no consensus on the optimal methods for measuring lipophilic chemicals in serum. The use of serum BFRs concentrations as markers of exposure in this study is appropriate given their widespread application in environmental health research. However, serum measurements have limitations in capturing long-term or cumulative exposure, particularly for lipophilic and bioaccumulative compounds like BFRs. While serum levels reflect recent or circulating exposure, they may not fully account for the total body burden of BFRs, which can be stored in adipose tissue and other compartments over time. Alternative approaches, such as measuring BFRs concentrations in fat biopsies, breast milk, or hair, could provide more comprehensive assessments of cumulative exposure but were not feasible within the constraints of this study. Future research should consider integrating these biomarkers to better capture the full scope of BFRs exposure. Third, the pathogenic characteristics and mechanisms of BFRs in both animals and humans remain unclear. Additionally, the long-term accumulation effects of BFRs in the body may require extended follow-up periods for a comprehensive assessment.

Overall, our study demonstrates a significant association between BFRs exposure and PhenoAgeAccel, revealing gender differences in exposure effects. This finding offers new insights into how environmental pollutants can influence biological aging. These results underscore the importance of public health interventions aimed at reducing BFRs exposure. Moreover, given the greater susceptibility observed in males, targeted exposure reduction strategies—such as regulatory restrictions, workplace safety measures, and public awareness—are essential to mitigate potential health risks. Future research should include more longitudinal studies to determine the causal relationship between BFRs exposure and aging and validate these findings in broader populations, while also exploring the underlying mechanisms involved.

## Data Availability

Publicly available datasets were analyzed in this study. This data can be found at: nhanes: https://wwwn.cdc.gov/nchs/nhanes/Default.aspx.
