# Supplementary material for: Sex-specific associations between brominated flame retardants exposure and phenotypic age acceleration in NHANES 2005–2010
Source: Front Public Health. 2025 Apr 8;13:1513757. doi: 10.3389/fpubh.2025.1513757 (PMC12011723; doi:10.3389/fpubh.2025.1513757)
Supplement: Supplementary file 1 [file Table_1.docx]

**Online Supplementary Material**


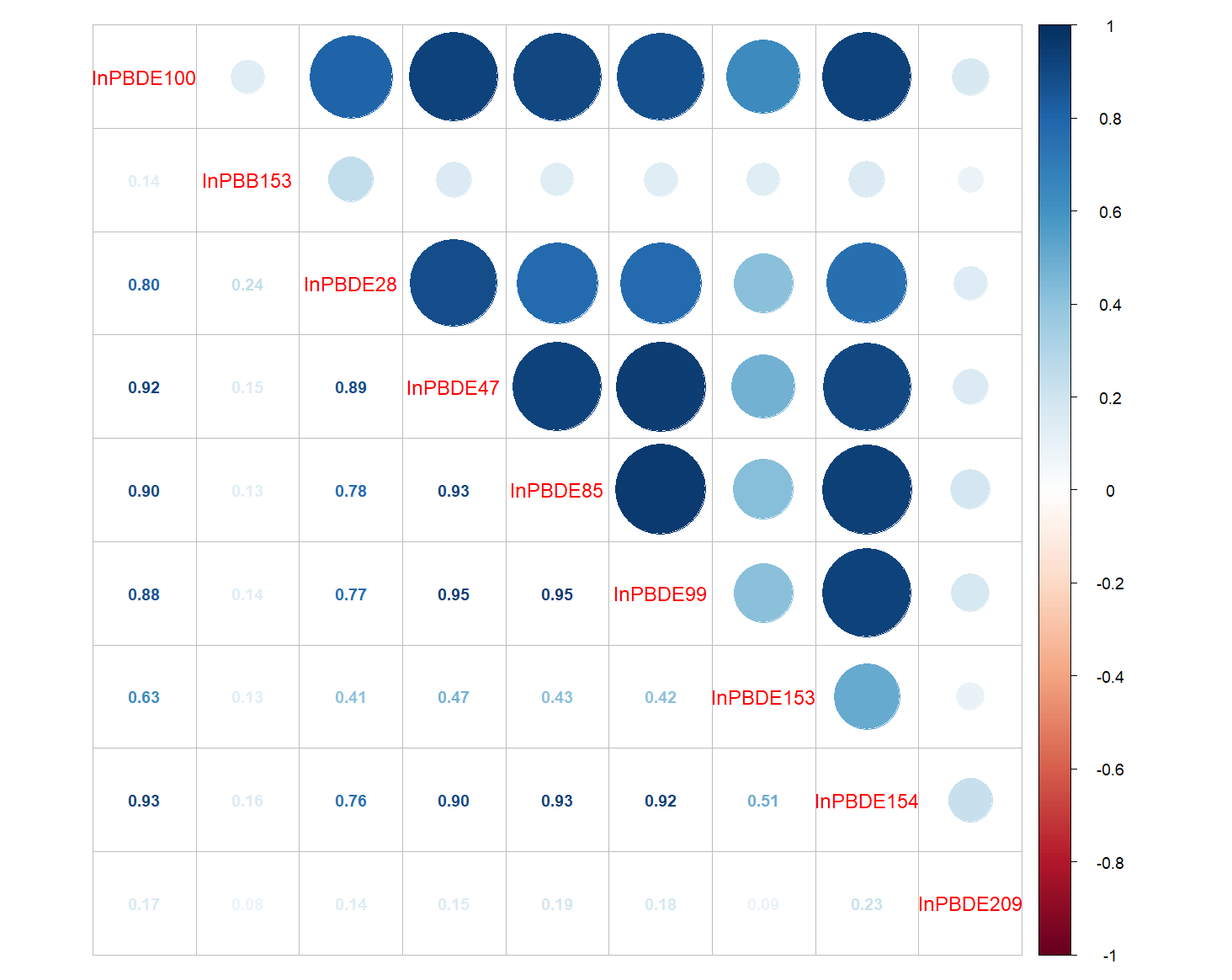


**Figure S1.** Spearman Analysis of the nine Substances (Brominated Flame Retardants)


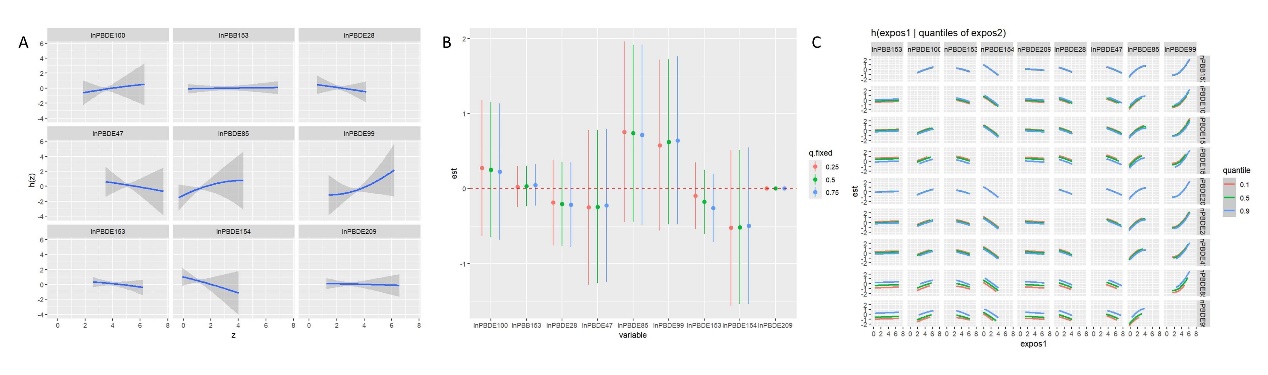


**Figure S2.** Associations between BFRs mixture and PhenoAgeAccel by Bayesian kernel machine regression (BKMR) for adults in NHANES 2005 -2010. A: Joint effect of perchlorate, nitrate, and thiocyanate mixture on PhenoAgeAccel calculated by the BKMR model. B: Single chemical-exposure effect (95% CI) to PhenoAgeAccel when other chemicals were fixed at a specific quantile (25th, 50th, 75th). C: Bivariate exposure-response relationship between three anions and risk of PhenoAgeAccel (a visualization for evaluating interactions). These models were adjusted for age, sex, ethnicity, BMI, PIR, marital status, education, physical activity, smoke, drinks, Hypertension, DM, CVD, cancer, eGFR, HEI-2015, and Year.

**Table S1.** Characteristics of the study participants among U.S adults by sex

(NHANES 2005-2010).

| **variable** | **Total (n=3908)** | **Male (n=1987)** | **Female (n=1921)** | P value |
| --- | --- | --- | --- | --- |
| **Phenotypic age** | 46.22 ± 20.66 | 46.32 ± 21.31 | 46.12 ± 19.98 | 0.77 |
| **PhenoAgeAccel** | -3.23 ± 8.40 | -2.78 ± 8.14 | -3.70 ± 8.64 | <0.001 |
| **PhenoAgeAccel** |  |  |  | 0.29 |
| Delayed | 3004(76.87) | 1513(76.14) | 1491(77.62) |  |
| Accelerated | 904(23.13) | 474(23.86) | 430(22.38) |  |
| **Age** | 49.45 ± 17.66 | 49.10 ± 17.83 | 49.82 ± 17.47 | 0.20 |
| **Age group** |  |  |  | 0.09 |
| <40 | 1310(33.52) | 698(35.13) | 612(31.86) |  |
| 40-59 | 1293(33.09) | 647(32.56) | 646(33.63) |  |
| ≥60 | 1305(33.39) | 642(32.31) | 663(34.51) |  |
| **Ethnicity** |  |  |  | 0.81 |
| Non-Hispanic White | 1971(50.44) | 995(50.08) | 976(50.81) |  |
| Non-Hispanic Black | 737(18.86) | 370(18.62) | 367(19.10) |  |
| Mexican American | 709(18.14) | 372(18.72) | 337(17.54) |  |
| Others | 491(12.56) | 250(12.58) | 241(12.55) |  |
| **PIR** |  |  |  | 0.08 |
| Low | 1096(28.05) | 533(26.82) | 563(29.31) |  |
| Middle | 1553(39.74) | 784(39.46) | 769(40.03) |  |
| High | 1259(32.22) | 670(33.72) | 589(30.66) |  |
| **BMI** |  |  |  | <.001 |
| Obesity | 1438(36.80) | 672(33.82) | 766(39.88) |  |
| Normal | 1102(28.20) | 512(25.77) | 590(30.71) |  |
| Overweight | 1368(35.01) | 803(40.41) | 565(29.41) |  |
| **Marital status** |  |  |  | <.001 |
| Never married | 613(15.69) | 331(16.66) | 282(14.68) |  |
| Widowed/Divorced/Separated | 885(22.65) | 322(16.21) | 563(29.31) |  |
| Married/Living with partner | 2410(61.67) | 1334(67.14) | 1076(56.01) |  |
| **Education** |  |  |  | 0.02 |
| Middle school or lower | 453(11.59) | 244(12.28) | 209(10.88) |  |
| High school | 1537(39.33) | 812(40.87) | 725(37.74) |  |
| College or more | 1918(49.08) | 931(46.85) | 987(51.38) |  |
| **Physical activity** |  |  |  | <.001 |
| Inactive | 910(23.29) | 429(21.59) | 481(25.04) |  |
| Moderate | 480(12.28) | 220(11.07) | 260(13.53) |  |
| Active | 1520(38.89) | 924(46.50) | 596(31.03) |  |
| Others | 998(25.54) | 414(20.84) | 584(30.40) |  |
| **Smoke** |  |  |  | <.001 |
| Never | 2040(52.20) | 871(43.83) | 1169(60.85) |  |
| Former | 1033(26.43) | 645(32.46) | 388(20.20) |  |
| Now | 835(21.37) | 471(23.70) | 364(18.95) |  |
| **Drinks** |  |  |  | <.001 |
| Former | 720(18.42) | 366(18.42) | 354(18.43) |  |
| Mild | 1257(32.16) | 739(37.19) | 518(26.97) |  |
| Never | 507(12.97) | 132(6.64) | 375(19.52) |  |
| Moderate | 561(14.36) | 220(11.07) | 341(17.75) |  |
| Heavy | 863(22.08) | 530(26.67) | 333(17.33) |  |
| **Hypertension** | 1590(40.69) | 813(40.92) | 777(40.45) | 0.79 |
| **DM** | 675(17.27) | 354(17.82) | 321(16.71) | 0.38 |
| **CVD** | 286(7.32) | 174(8.76) | 112(5.83) | <0.001 |
| **Cancer** | 369(9.44) | 174(8.76) | 195(10.15) | 0.15 |
| **HEI-2015** | 50.62 ± 13.49 | 49.66 ± 13.01 | 51.62 ± 13.91 | <.001 |
| **eGFR** | 93.14 ± 23.31 | 93.09 ± 22.20 | 93.19 ± 24.42 | 0.89 |
| **PBB153*** | 37.99 ± 69.69 | 46.53 ± 88.28 | 29.17 ± 40.84 | <.001 |
| **PBDE28*** | 11.20 ± 7.50 | 11.49 ± 7.30 | 10.91 ± 7.69 | 0.02 |
| **PBDE47*** | 219.52 ± 204.75 | 233.77 ± 195.37 | 204.78 ± 213.07 | <.001 |
| **PBDE85*** | 4.94 ± 5.96 | 5.16 ± 5.05 | 4.72 ± 6.77 | 0.02 |
| **PBDE99*** | 49.70 ± 61.06 | 54.50 ± 64.39 | 44.75 ± 57.01 | <.001 |
| **PBDE100*** | 45.24 ± 44.26 | 48.47 ± 40.27 | 41.90 ± 47.83 | <.001 |
| **PBDE153*** | 76.61 ± 67.71 | 89.30 ± 77.18 | 63.48 ± 53.19 | <.001 |
| **PBDE154*** | 4.51 ± 4.86 | 4.86 ± 4.54 | 4.15 ± 5.14 | <.001 |
| **PBDE209*** | 22.99 ± 39.75 | 24.88 ± 38.16 | 21.04 ± 41.25 | <0.01 |

Continuous variable was presented as mean ± SD and categorical variables were presented as numbers (percentages). Variables between groups were compared by Student’s t-tests and chi-square tests

*Unit: pg/g.

**Table S2.** Characteristics of the study participants of accelerated phenotypic age among U.S adults by sex (NHANES 2005-2010).

| **Variable** | **Total (n=904)** | **Male (n=474)** | **Female (n=430)** | **P value** |
| --- | --- | --- | --- | --- |
| **Phenotypic age** | 62.58 ± 20.96 | 63.97 ± 20.95 | 61.05 ± 20.90 | 0.04 |
| **PhenoAgeAccel** | 7.80 ± 10.04 | 7.46 ± 10.19 | 8.18 ± 9.87 | 0.27 |
| **Age** | 54.78 ± 17.46 | 56.51 ± 16.97 | 52.87 ± 17.81 | <0.01 |
| **Age group** |  |  |  | 0.01 |
| <40 | 211(23.34) | 96(20.25) | 115(26.74) |  |
| 40-59 | 415(45.91) | 238(50.21) | 177(41.16) |  |
| ≥60 | 278(30.75) | 140(29.54) | 138(32.09) |  |
| **Ethnicity** |  |  |  | <0.01 |
| Non-Hispanic White | 402(44.47) | 233(49.16) | 169(39.30) |  |
| Non-Hispanic Black | 245(27.10) | 126(26.58) | 119(27.67) |  |
| Mexican American | 165(18.25) | 78(16.46) | 87(20.23) |  |
| Others | 92(10.18) | 37( 7.81) | 55(12.79) |  |
| **PIR** |  |  |  | 0.15 |
| Low | 333(36.84) | 162(34.18) | 171(39.77) |  |
| Middle | 366(40.49) | 195(41.14) | 171(39.77) |  |
| High | 205(22.68) | 117(24.68) | 88(20.47) |  |
| **BMI** |  |  |  | <.001 |
| Obesity | 497(54.98) | 226(47.68) | 271(63.02) |  |
| Normal | 161(17.81) | 88(18.57) | 73(16.98) |  |
| Overweight | 246(27.21) | 160(33.76) | 86(20.00) |  |
| **Marital status** |  |  |  | <.001 |
| Never married | 115(12.72) | 61(12.87) | 54(12.56) |  |
| Widowed/Divorced/Separated | 284(31.42) | 119(25.11) | 165(38.37) |  |
| Married/Living with partner | 505(55.86) | 294(62.03) | 211(49.07) |  |
| **Education** |  |  |  | 0.34 |
| Middle school or lower | 136(15.04) | 71(14.98) | 65(15.12) |  |
| High school | 398(44.03) | 219(46.20) | 179(41.63) |  |
| College or more | 370(40.93) | 184(38.82) | 186(43.26) |  |
| **Physical activity** |  |  |  | 0.02 |
| Inactive | 198(21.90) | 108(22.78) | 90(20.93) |  |
| Moderate | 89(9.85) | 43(9.07) | 46(10.70) |  |
| Active | 296(32.74) | 174(36.71) | 122(28.37) |  |
| Others | 321(35.51) | 149(31.43) | 172(40.00) |  |
| **Smoke** |  |  |  | <.001 |
| Never | 392(43.36) | 160(33.76) | 232(53.95) |  |
| Former | 267(29.54) | 176(37.13) | 91(21.16) |  |
| Now | 245(27.10) | 138(29.11) | 107(24.88) |  |
| **Drinks** |  |  |  | <.001 |
| Former | 241(26.66) | 130(27.43) | 111(25.81) |  |
| Mild | 247(27.32) | 157(33.12) | 90(20.93) |  |
| Never | 131(14.49) | 35(7.38) | 96(22.33) |  |
| Moderate | 103(11.39) | 43(9.07) | 60(13.95) |  |
| Heavy | 182(20.13) | 109(23.00) | 73(16.98) |  |
| **Hypertension** | 530(58.63) | 273(57.59) | 257(59.77) | 0.55 |
| **DM** | 382(42.26) | 202(42.62) | 180(41.86) | 0.87 |
| **CVD** | 130(14.38) | 86(18.14) | 44(10.23) | <0.01 |
| **Cancer** | 122(13.50) | 71(14.98) | 51(11.86) | 0.20 |
| **HEI-2015** | 49.41 ± 13.21 | 48.70 ± 12.94 | 50.20 ± 13.49 | 0.09 |
| **eGFR** | 83.02 ± 28.16 | 81.13 ± 25.96 | 85.10 ± 30.30 | 0.04 |
| **PBB153*** | 40.26 ± 66.76 | 49.88 ± 83.72 | 29.66 ± 37.93 | <.001 |
| **PBDE28*** | 12.08 ± 7.85 | 12.41 ± 7.28 | 11.71 ± 8.42 | 0.19 |
| **PBDE47*** | 242.58 ± 230.29 | 260.69 ± 216.58 | 222.61 ± 243.20 | 0.01 |
| **PBDE85*** | 5.63 ± 6.92 | 5.82 ± 5.58 | 5.41 ± 8.14 | 0.37 |
| **PBDE99*** | 57.72 ± 72.73 | 65.48 ± 81.10 | 49.16 ± 61.17 | <0.001 |
| **PBDE100*** | 48.70 ± 48.60 | 51.46 ± 40.27 | 45.65 ± 56.26 | 0.08 |
| **PBDE153*** | 73.20 ± 62.03 | 83.07 ± 68.49 | 62.32 ± 51.97 | <.001 |
| **PBDE154*** | 5.01 ± 5.55 | 5.44 ± 5.16 | 4.54 ± 5.92 | 0.02 |
| **PBDE209*** | 23.85 ± 44.90 | 26.30 ± 50.29 | 21.15 ± 37.95 | 0.08 |

Continuous variable was presented as mean ± SD and categorical variables were presented as numbers (percentages). Variables between groups were compared by Student’s t-tests and chi-square tests

*Unit: pg/g.

**Table S3:** Subgroup and interaction analyses of BFRs exposures and PhenoAgeAccel, stratified by age group and sex.

| Character | β (95% CI) | P value | P for interaction |  |
| --- | --- | --- | --- | --- |
| **PBB153** |  |  |  |  |
| Age group |  |  | 0.289 |  |
| <40 | 0.044(-0.336, 0.424) | 0.821 |  |  |
| 40-59 | 0.141(-0.335, 0.617) | 0.561 |  |  |
| ≥60 | 0.055(-0.591, 0.701) | 0.867 |  |  |
| Sex |  |  | < .001 |  |
| Male | -0.325(-0.675, 0.025) | 0.068 |  |  |
| Female | -0.633(-1.051, -0.216) | 0.003 |  |  |
| **PBDE28** |  |  |  |  |
| Age group |  |  | 0.339 |  |
| <40 | 0.194(-0.399, 0.788) | 0.521 |  |  |
| 40-59 | 0.219(-0.605, 1.044) | 0.602 |  |  |
| ≥60 | 0.983(0.224, 1.742) | 0.011 |  |  |
| Sex |  |  | 0.002 |  |
| Male | 0.64(0.078, 1.203) | 0.026 |  |  |
| Female | -0.186(-0.808, 0.436) | 0.558 |  |  |
| **PBDE85** |  |  |  |  |
| Age group |  |  | 0.182 |  |
| <40 | 0.155(-0.341, 0.651) | 0.541 |  |  |
| 40-59 | 0.011(-0.682, 0.705) | 0.975 |  |  |
| ≥60 | 0.906(0.252, 1.560) | 0.007 |  |  |
| Sex |  |  | 0.003 |  |
| Male | 0.699(0.218, 1.181) | 0.004 |  |  |
| Female | -0.207(-0.746, 0.332) | 0.452 |  |  |
| **PBDE47** |  |  |  |  |
| Age group |  |  | 0.27 |  |
| <40 | 0.328(-0.111, 0.767) | 0.143 |  |  |
| 40-59 | | 0.071(-0.541, 0.682) | 0.821 |  |
| ≥60 | 0.822(0.232, 1.412) | 0.006 |  |  |
| Sex |  |  | 0.017 |  |
| Male | 0.636(0.209, 1.064) | 0.004 |  |  |
| Female | -0.092(-0.574, 0.390) | 0.708 |  |  |
| **PBDE99** |  |  |  |  |
| Age group |  |  | 0.027 |  |
| <40 | 0.316(-0.110, 0.742) | 0.145 |  |  |
| 40-59 | -0.229(-0.819, 0.360) | 0.446 |  |  |
| ≥60 | 0.91(0.348, 1.473) | 0.002 |  |  |
| Sex |  |  | 0.003 |  |
| Male | 0.666(0.261, 1.071) | 0.001 |  |  |
| Female | -0.199(-0.676, 0.277) | 0.412 |  |  |
| **PBDE100** |  |  |  |  |
| Age group |  |  | 0.042 |  |
| <40 | -0.041(-0.514, 0.432) | 0.864 |  |  |
| 40-59 | 0.5(-0.089, 1.088) | 0.096 |  |  |
| ≥60 | 0.549(-0.035, 1.134) | 0.065 |  |  |
| Sex |  |  | 0.148 |  |
| Male | 0.022(-0.416, 0.461) | 0.920 |  |  |
| Female | 0.611(0.117, 1.106) | 0.015 |  |  |
| **PBDE154** |  |  |  |  |
| Age group |  |  | 0.347 |  |
| <40 | 0.242(-0.233, 0.717) | 0.317 |  |  |
| 40-59 | 0.088(-0.542, 0.718) | 0.785 |  |  |
| ≥60 | | 0.681(0.057, 1.305) | 0.033 |  |
| Sex |  |  | 0.025 |  |
| Male | 0.513(0.072, 0.954) | 0.023 |  |  |
| Female | -0.15(-0.675, 0.375) | 0.575 |  |  |
| **PBDE209** |  |  |  |  |
| Age group |  |  | 0.005 |  |
| <40 | -0.061(-0.708, 0.585) | 0.853 |  |  |
| 40-59 | 1.138(0.371, 1.905) | 0.004 |  |  |
| ≥60 | | -0.481(-1.421, 0.459) | 0.316 |  |
| Sex |  |  | 0.725 |  |
| Male | 0.289(-0.295, 0.873) | 0.332 |  |  |
| Female | -0.206(-0.975, 0.563) | 0.599 |  |  |
| **PBDE153** |  |  |  |  |
| Age group |  |  | 0.577 |  |
| <40 | 0.085(-0.395, 0.566) | 0.727 |  |  |
| 40-59 | 0.249(-0.398, 0.896) | 0.451 |  |  |
| ≥60 | | 0.624(0.028, 1.220) | 0.040 |  |
| Sex |  |  | 0.1 |  |
| Male | 0.478(0.032, 0.925) | 0.036 |  |  |
| Female | 0.007(-0.505, 0.519) | 0.979 |  |  |

Models adjusted for age, sex, ethnicity, BMI, PIR, marital status, education, physical activity, smoke, drinks, Hypertension, DM, CVD, cancer, eGFR, HEI-2015, and Year. The subgroup variable was not included in same subgroup analysis.
