# Supplementary material for: Sex-specific associations between brominated flame retardants exposure and phenotypic age acceleration in NHANES 2005–2010
Source: Front Public Health. 2025 Apr 8;13:1513757. doi: 10.3389/fpubh.2025.1513757 (PMC12011723; doi:10.3389/fpubh.2025.1513757)
Supplement: Supplementary file 3 [file Table_3.docx]

library(charlsR)# 收费的R包，数据向量化，Table 1 ，多元回归,分层分析，交互作用检验及RCS使用了CharlsR里面的函数，可以由其它R包替代。

library(reshape2)

library(do)

library(dplyr)

library(openxlsx)

library('mice')

library(aod)

library(survey)

library(rms)

library(bkmr)

library(corrplot)

library("ggplot2") #加载这些包#

library(gWQS)

library(ggplot2)

library(epiDisplay)

#N=31034,大于20 17132, 除去BFRs留下 4971,除去aing 留下 4926, 除去协变量缺失 留下 3908；

#不加权的可以用CharlsR====

subd <- read.xlsx("C:\\Users\\Livek\\Desktop\\NHANES\\【2024】\\env05\\溴化物衰老(F02).xlsx")

# Factor(subd)

subd$Year <- factor(subd$Year, c('2005-2006', '2007-2008', '2009-2010'))

subd$age2 <- factor(subd$age2, c('<20', '≥20'))

subd$age3 <- factor(subd$age3, c('<40', '≥60', '40-59'))

subd$sex <- factor(subd$sex, c('Male', 'Female'))

subd$ethnicity <- factor(subd$ethnicity, c('Non-Hispanic White', 'Non-Hispanic Black', 'Mexican American', 'others'))

subd$poverty3 <- factor(subd$poverty3, c('low', 'middle', 'high'))

subd$bmi3 <- factor(subd$bmi3, c('obesity', 'normal', 'overweight'))

subd$marital3 <- factor(subd$marital3, c('Never married', 'WidowedDivorcedSeparated', 'Married/Living with partner'))

subd$home2 <- factor(subd$home2, c('rented or others', 'owned or being bought'))

subd$education <- factor(subd$education, c('Middle school or lower', 'High school', 'College or more'))

subd$pa3 <- factor(subd$pa3, c( 'inactive', 'moderate', 'active','others'))

subd$smoke <- factor(subd$smoke, c('never', 'former', 'now'))

subd$drinks <- factor(subd$drinks, c('former', 'mild', 'never', 'moderate', 'heavy'))

subd$Hypertension <- factor(subd$Hypertension, c('no', 'yes'))

subd$DM <- factor(subd$DM, c('no', 'yes'))

subd$cvd <- factor(subd$cvd, c('no', 'yes'))

subd$cancer <- factor(subd$cancer, c('no', 'yes'))

subd$aging <- factor(subd$aging, levels = c("Delayed","Accelerated"))

d2 <- subset(subd,subd$select=="1")

d4 <- subset(d2,d2$aging=="Accelerated")

#Table 01====

# table_1(d2)

table_1(data=d2,

# 指定连续变量

lx.z=c('seqn','age','hei2015_total_score','CKD_EPI_Scr_2009',

'PBB153','PBDE28','PBDE47','PBDE85','PBDE99','PBDE100','PBDE153','PBDE154','PBDE209',

'phynotypicage','PhenoAgeAccel'),

lx.fz=NULL,

# 指定分类变量

fl=c('aging',"age3" ,"sex",'ethnicity','poverty3','bmi3','marital3','home2','education','pa3','smoke','drinks','Hypertension','DM','cvd','cancer'),

direction = 'v', # 指定分类变量计算的方向

# by= "aging", # 指定分组变量

# 变量展示顺序

showOrder=NULL,

# 如果需要做交互作用

time=NULL,

y=NULL,

adjust=NULL,

# 如果需要把结果输出到excel中

xlsx="C:\\Users\\Livek\\Desktop\\tableall.xlsx",

round=2)

table_1(data=d2,

# 指定连续变量

lx.z=c('seqn','age','hei2015_total_score','CKD_EPI_Scr_2009',

'PBB153','PBDE28','PBDE47','PBDE85','PBDE99','PBDE100','PBDE153','PBDE154','PBDE209',

'phynotypicage','PhenoAgeAccel'),

lx.fz=NULL,

# 指定分类变量

fl=c('aging',"age3" ,"sex",'ethnicity','poverty3','bmi3','marital3','home2','education','pa3','smoke','drinks','Hypertension','DM','cvd','cancer'),

direction = 'v', # 指定分类变量计算的方向

by="aging", # 指定分组变量

# 变量展示顺序

showOrder=NULL,

# 如果需要做交互作用

time=NULL,

y=NULL,

adjust=NULL,

# 如果需要把结果输出到excel中

xlsx="C:\\Users\\Livek\\Desktop\\table.xlsx",

round=2)

table_1(data=d2,

# 指定连续变量

lx.z=c('seqn','age','hei2015_total_score','CKD_EPI_Scr_2009',

'PBB153','PBDE28','PBDE47','PBDE85','PBDE99','PBDE100','PBDE153','PBDE154','PBDE209',

'phynotypicage','PhenoAgeAccel'),

lx.fz=NULL,

# 指定分类变量

fl=c("age3" ,"sex",'ethnicity','poverty3','bmi3','marital3','home2','education','pa3','smoke','drinks','Hypertension','DM','cvd','cancer'),

direction = 'v', # 指定分类变量计算的方向

by="sex", # 指定分组变量

# 变量展示顺序

showOrder=NULL,

# 如果需要做交互作用

time=NULL,

y=NULL,

adjust=NULL,

# 如果需要把结果输出到excel中

xlsx="C:\\Users\\Livek\\Desktop\\tablesexS02.xlsx",

round=2)

table_1(data=d4,

# 指定连续变量

lx.z=c('seqn','age','hei2015_total_score','CKD_EPI_Scr_2009',

'PBB153','PBDE28','PBDE47','PBDE85','PBDE99','PBDE100','PBDE153','PBDE154','PBDE209',

'phynotypicage','PhenoAgeAccel'),

lx.fz=NULL,

# 指定分类变量

fl=c("age3" ,"sex",'ethnicity','poverty3','bmi3','marital3','home2','education','pa3','smoke','drinks','Hypertension','DM','cvd','cancer'),

direction = 'v', # 指定分类变量计算的方向

by="sex", # 指定分组变量

# 变量展示顺序

showOrder=NULL,

# 如果需要做交互作用

time=NULL,

y=NULL,

adjust=NULL,

# 如果需要把结果输出到excel中

xlsx="C:\\Users\\Livek\\Desktop\\tableAcceleratedS02.xlsx",

round=2)

#Table 02====

a1 <-ols(PhenoAgeAccel ~ lnPBB153 ,data = d2)%>% reg_table(2)

a2 <-ols(PhenoAgeAccel ~ lnPBDE28 ,data = d2)%>% reg_table(2)

a3 <-ols(PhenoAgeAccel ~ lnPBDE47 ,data = d2)%>% reg_table(2)

a4 <-ols(PhenoAgeAccel ~ lnPBDE85 ,data = d2)%>% reg_table(2)

a5 <-ols(PhenoAgeAccel ~ lnPBDE99 ,data = d2)%>% reg_table(2)

a6 <-ols(PhenoAgeAccel ~ lnPBDE100 ,data = d2)%>% reg_table(2)

a7 <-ols(PhenoAgeAccel ~ lnPBDE154 ,data = d2)%>% reg_table(2)

a8 <-ols(PhenoAgeAccel ~ lnPBDE209 ,data = d2)%>% reg_table(2)

a9 <-ols(PhenoAgeAccel ~ lnPBDE153 ,data = d2)%>% reg_table(2)

a10 <-ols(PhenoAgeAccel ~ lnPBB153 +age+sex+ethnicity,data = d2)%>% reg_table(2)

a20 <-ols(PhenoAgeAccel ~ lnPBDE28 +age+sex+ethnicity,data = d2)%>% reg_table(2)

a30 <-ols(PhenoAgeAccel ~ lnPBDE47 +age+sex+ethnicity,data = d2)%>% reg_table(2)

a40 <-ols(PhenoAgeAccel ~ lnPBDE85 +age+sex+ethnicity,data = d2)%>% reg_table(2)

a50 <-ols(PhenoAgeAccel ~ lnPBDE99 +age+sex+ethnicity,data = d2)%>% reg_table(2)

a60 <-ols(PhenoAgeAccel ~ lnPBDE100 +age+sex+ethnicity,data = d2)%>% reg_table(2)

a70 <-ols(PhenoAgeAccel ~ lnPBDE154 +age+sex+ethnicity,data = d2)%>% reg_table(2)

a80 <-ols(PhenoAgeAccel ~ lnPBDE209 +age+sex+ethnicity,data = d2)%>% reg_table(2)

a90 <-ols(PhenoAgeAccel ~ lnPBDE153 +age+sex+ethnicity,data = d2)%>% reg_table(2)

a11 <-ols(PhenoAgeAccel ~ lnPBB153 +age+sex+ethnicity+poverty3+BMI+marital3+home2+education+pa3+smoke+drinks+Hypertension+ DM+ cvd+ cancer+hei2015_total_score+Year+CKD_EPI_Scr_2009,data = d2)%>% reg_table(2)

a21 <-ols(PhenoAgeAccel ~ lnPBDE28 +age+sex+ethnicity+poverty3+BMI+marital3+home2+education+pa3+smoke+drinks+Hypertension+ DM+ cvd+ cancer+hei2015_total_score+Year+CKD_EPI_Scr_2009,data = d2)%>% reg_table(2)

a31 <-ols(PhenoAgeAccel ~ lnPBDE47 +age+sex+ethnicity+poverty3+BMI+marital3+home2+education+pa3+smoke+drinks+Hypertension+ DM+ cvd+ cancer+hei2015_total_score+Year+CKD_EPI_Scr_2009,data = d2)%>% reg_table(2)

a41 <-ols(PhenoAgeAccel ~ lnPBDE85 +age+sex+ethnicity+poverty3+BMI+marital3+home2+education+pa3+smoke+drinks+Hypertension+ DM+ cvd+ cancer+hei2015_total_score+Year+CKD_EPI_Scr_2009,data = d2)%>% reg_table(2)

a51 <-ols(PhenoAgeAccel ~ lnPBDE99 +age+sex+ethnicity+poverty3+BMI+marital3+home2+education+pa3+smoke+drinks+Hypertension+ DM+ cvd+ cancer+hei2015_total_score+Year+CKD_EPI_Scr_2009,data = d2)%>% reg_table(2)

a61 <-ols(PhenoAgeAccel ~ lnPBDE100 +age+sex+ethnicity+poverty3+BMI+marital3+home2+education+pa3+smoke+drinks+Hypertension+ DM+ cvd+ cancer+hei2015_total_score+Year+CKD_EPI_Scr_2009,data = d2)%>% reg_table(2)

a71 <-ols(PhenoAgeAccel ~ lnPBDE154 +age+sex+ethnicity+poverty3+BMI+marital3+home2+education+pa3+smoke+drinks+Hypertension+ DM+ cvd+ cancer+hei2015_total_score+Year+CKD_EPI_Scr_2009,data = d2)%>% reg_table(2)

a81 <-ols(PhenoAgeAccel ~ lnPBDE209 +age+sex+ethnicity+poverty3+BMI+marital3+home2+education+pa3+smoke+drinks+Hypertension+ DM+ cvd+ cancer+hei2015_total_score+Year+CKD_EPI_Scr_2009,data = d2)%>% reg_table(2)

a91 <-ols(PhenoAgeAccel ~ lnPBDE153 +age+sex+ethnicity+poverty3+BMI+marital3+home2+education+pa3+smoke+drinks+Hypertension+ DM+ cvd+ cancer+hei2015_total_score+Year+CKD_EPI_Scr_2009,data = d2)%>% reg_table(2)

crude.Model.n(a1,a10,a11,

a2,a20,a21,

a3,a30,a31,

a4,a40,a41,

a5,a50,a51,

a6,a60,a61,

a7,a70,a71,

a8,a80,a81,

a9,a90,a91,xlsx="C:\\Users\\Livek\\Desktop\\BPD衰老加速\\table2.xlsx")

#Figure 03 RCS----

#PhenoAgeAccel

f1 <-ols(PhenoAgeAccel ~ rcs(lnPBB153,3) +age+sex+ethnicity+poverty3+BMI+marital3+home2+education+pa3+smoke+Hypertension+ DM+ cvd+ cancer+hei2015_total_score+Year+CKD_EPI_Scr_2009,d2)

f2 <-ols(PhenoAgeAccel ~ rcs(lnPBDE28,3) +age+sex+ethnicity+poverty3+BMI+marital3+home2+education+pa3+smoke+Hypertension+ DM+ cvd+ cancer+hei2015_total_score+Year+CKD_EPI_Scr_2009,d2)

f3 <-ols(PhenoAgeAccel ~ rcs(lnPBDE47 ,3) +age+sex+ethnicity+poverty3+BMI+marital3+home2+education+pa3+smoke+Hypertension+ DM+ cvd+ cancer+hei2015_total_score+Year+CKD_EPI_Scr_2009,d2)

f4 <-ols(PhenoAgeAccel ~ rcs(lnPBDE85 ,3) +age+sex+ethnicity+poverty3+BMI+marital3+home2+education+pa3+smoke+Hypertension+ DM+ cvd+ cancer+hei2015_total_score+Year+CKD_EPI_Scr_2009,d2)

f5 <-ols(PhenoAgeAccel ~ rcs(lnPBDE99 ,3) +age+sex+ethnicity+poverty3+BMI+marital3+home2+education+pa3+smoke+Hypertension+ DM+ cvd+ cancer+hei2015_total_score+Year+CKD_EPI_Scr_2009,d2)

f6 <-ols(PhenoAgeAccel ~ rcs(lnPBDE100 ,3) +age+sex+ethnicity+poverty3+BMI+marital3+home2+education+pa3+smoke+Hypertension+ DM+ cvd+ cancer+hei2015_total_score+Year+CKD_EPI_Scr_2009,d2)

f7 <-ols(PhenoAgeAccel ~ rcs(lnPBDE154 ,3) +age+sex+ethnicity+poverty3+BMI+marital3+home2+education+pa3+smoke+Hypertension+ DM+ cvd+ cancer+hei2015_total_score+Year+CKD_EPI_Scr_2009,d2)

f8 <-ols(PhenoAgeAccel ~ rcs(lnPBDE209 ,3) +age+sex+ethnicity+poverty3+BMI+marital3+home2+education+pa3+smoke+Hypertension+ DM+ cvd+ cancer+hei2015_total_score+Year+CKD_EPI_Scr_2009,d2)

f9 <-ols(PhenoAgeAccel ~ rcs(lnPBDE153 ,3) +age+sex+ethnicity+poverty3+BMI+marital3+home2+education+pa3+smoke+Hypertension+ DM+ cvd+ cancer+hei2015_total_score+Year+CKD_EPI_Scr_2009,d2)

r1 <- RCS(f1)

r2 <- RCS(f2)

r3 <- RCS(f3)

r4 <- RCS(f4)

r5 <- RCS(f5)

r6 <- RCS(f6)

r7 <- RCS(f7)

r8 <- RCS(f8)

r9 <- RCS(f9)

rcs_plot1.1(r1,reference=F,

title = "A",

xlab = "PBB153(log transformed)",

ylab = "PhenoAgeAccel",

lp.color = "red",

ci.alpha = 0.3,

file = "C:\\Users\\Livek\\Desktop\\BPD衰老加速\\数据集\\prcs01.jpg",

width = 7,height = 6)

rcs_plot1.1(r2,reference=F,

title = "A",

xlab = "PBDE28(log transformed)",

ylab = "PhenoAgeAccel",

lp.color = "red",

ci.alpha = 0.3,

file = "C:\\Users\\Livek\\Desktop\\BPD衰老加速\\数据集\\prcs02.jpg",

width = 7,height = 6)

rcs_plot1.1(r3,reference=F,

title = "A",

xlab = "PBDE47(log transformed)",

ylab = "PhenoAgeAccel",

lp.color = "red",

ci.alpha = 0.3,

file = "C:\\Users\\Livek\\Desktop\\BPD衰老加速\\数据集\\prcs03.jpg",

width = 7,height = 6)

rcs_plot1.1(r4,reference=F,

title = "A",

xlab = "PBDE85(log transformed)",

ylab = "PhenoAgeAccel",

lp.color = "red",

ci.alpha = 0.3,

file = "C:\\Users\\Livek\\Desktop\\BPD衰老加速\\数据集\\prcs04.jpg",

width = 7,height = 6)

rcs_plot1.1(r5,reference=F,

title = "A",

xlab = "PBDE99(log transformed)",

ylab = "PhenoAgeAccel",

lp.color = "red",

ci.alpha = 0.3,

file = "C:\\Users\\Livek\\Desktop\\BPD衰老加速\\数据集\\prcs05.jpg",

width = 7,height = 6)

rcs_plot1.1(r6,reference=F,

title = "A",

xlab = "PBDE100(log transformed)",

ylab = "PhenoAgeAccel",

lp.color = "red",

ci.alpha = 0.3,

file = "C:\\Users\\Livek\\Desktop\\BPD衰老加速\\数据集\\prcs06.jpg",

width = 7,height = 6)

rcs_plot1.1(r7,reference=F,

title = "A",

xlab = "PBDE154(log transformed)",

ylab = "PhenoAgeAccel",

lp.color = "red",

ci.alpha = 0.3,

file = "C:\\Users\\Livek\\Desktop\\BPD衰老加速\\数据集\\prcs07.jpg",

width = 7,height = 6)

rcs_plot1.1(r8,reference=F,

title = "A",

xlab = "PBDE209(log transformed)",

ylab = "PhenoAgeAccel",

lp.color = "red",

ci.alpha = 0.3,

file = "C:\\Users\\Livek\\Desktop\\BPD衰老加速\\数据集\\prcs08.jpg",

width = 7,height = 6)

rcs_plot1.1(r9,reference=F,

title = "A",

xlab = "PBDE153(log transformed)",

ylab = "PhenoAgeAccel",

lp.color = "red",

ci.alpha = 0.3,

file = "C:\\Users\\Livek\\Desktop\\BPD衰老加速\\数据集\\prcs09.jpg",

width = 7,height = 6)

#Table 03 by age3 and sex====

d2$dwqs <- d2$lnPBB153 * 0.242729328 +

d2$lnPBDE153 * 0.199349745 +

d2$lnPBDE28 * 0.198975723 +

d2$lnPBDE85 * 0.165685622 +

d2$lnPBDE209 * 0.076692233 +

d2$lnPBDE154 * 0.057348285 +

d2$lnPBDE99 * 0.034922331 +

d2$lnPBDE100 * 0.022347037 +

d2$lnPBDE47 * 0.001949698

p0<- stratum_model(data = d2,y="PhenoAgeAccel",x="dwqs",p= F,

stratum = c('sex'),

adjust =c('age3','ethnicity','poverty3','bmi3','marital3','home2',

'education','pa3','smoke','drinks','Hypertension','DM',

'cvd','cancer','hei2015_total_score','CKD_EPI_Scr_2009',"Year"))

p0<- stratum_model(data = d2,y="PhenoAgeAccel",x="dwqs",p= F,

stratum = c('age3'),

adjust =c('sex','ethnicity','poverty3','bmi3','marital3','home2',

'education','pa3','smoke','drinks','Hypertension','DM',

'cvd','cancer','hei2015_total_score','CKD_EPI_Scr_2009',"Year"))

p1<- stratum_model(data = d2,y="PhenoAgeAccel",x="lnPBB153",p= F,

stratum = c('age3','sex'),

adjust =c('ethnicity','poverty3','bmi3','marital3','home2',

'education','pa3','smoke','drinks','Hypertension','DM',

'cvd','cancer','hei2015_total_score','CKD_EPI_Scr_2009',"Year"),

, xlsx="C:\\Users\\Livek\\Desktop\\Table 03-1.xlsx")

p2 <- stratum_model(data = d2,y="PhenoAgeAccel",x="lnPBDE28",p= F,

stratum = c('age3','sex'),

adjust =c('ethnicity','poverty3','bmi3','marital3','home2',

'education','pa3','smoke','drinks','Hypertension','DM',

'cvd','cancer','hei2015_total_score','CKD_EPI_Scr_2009',"Year"),

, xlsx="C:\\Users\\Livek\\Desktop\\Table 03-2.xlsx")

p3 <- stratum_model(data = d2,y="PhenoAgeAccel",x="lnPBDE47",p= F,

stratum = c('age3','sex'),

adjust =c('ethnicity','poverty3','bmi3','marital3','home2',

'education','pa3','smoke','drinks','Hypertension','DM',

'cvd','cancer','hei2015_total_score','CKD_EPI_Scr_2009',"Year"),

, xlsx="C:\\Users\\Livek\\Desktop\\Table 03-3.xlsx")

P4 <- stratum_model(data = d2,y="PhenoAgeAccel",x="lnPBDE85",p= F,

stratum = c('age3','sex'),

adjust =c('ethnicity','poverty3','bmi3','marital3','home2',

'education','pa3','smoke','drinks','Hypertension','DM',

'cvd','cancer','hei2015_total_score','CKD_EPI_Scr_2009',"Year"),

, xlsx="C:\\Users\\Livek\\Desktop\\Table 03-4.xlsx")

p5 <- stratum_model(data = d2,y="PhenoAgeAccel",x="lnPBDE99",p= F,

stratum = c('age3','sex'),

adjust =c('ethnicity','poverty3','bmi3','marital3','home2',

'education','pa3','smoke','drinks','Hypertension','DM',

'cvd','cancer','hei2015_total_score','CKD_EPI_Scr_2009',"Year"),

, xlsx="C:\\Users\\Livek\\Desktop\\Table 03-5.xlsx")

P6 <- stratum_model(data = d2,y="PhenoAgeAccel",x="lnPBDE153",p= F,

stratum = c('age3','sex'),

adjust =c('ethnicity','poverty3','bmi3','marital3','home2',

'education','pa3','smoke','drinks','Hypertension','DM',

'cvd','cancer','hei2015_total_score','CKD_EPI_Scr_2009',"Year"),

, xlsx="C:\\Users\\Livek\\Desktop\\Table 03-6.xlsx")

p7 <- stratum_model(data = d2,y="PhenoAgeAccel",x="lnPBDE154",p= F,

stratum = c('age3','sex'),

adjust =c('ethnicity','poverty3','bmi3','marital3','home2',

'education','pa3','smoke','drinks','Hypertension','DM',

'cvd','cancer','hei2015_total_score','CKD_EPI_Scr_2009',"Year"),

, xlsx="C:\\Users\\Livek\\Desktop\\Table 03-7.xlsx")

P8 <- stratum_model(data = d2,y="PhenoAgeAccel",x="lnPBDE209",p= F,

stratum = c('age3','sex'),

adjust =c('ethnicity','poverty3','bmi3','marital3','home2',

'education','pa3','smoke','drinks','Hypertension','DM',

'cvd','cancer','hei2015_total_score','CKD_EPI_Scr_2009',"Year"),

, xlsx="C:\\Users\\Livek\\Desktop\\Table 03-8.xlsx")

P9 <- stratum_model(data = d2,y="PhenoAgeAccel",x="lnPBDE100",p= F,

stratum = c('age3','sex'),

adjust =c('ethnicity','poverty3','bmi3','marital3','home2',

'education','pa3','smoke','drinks','Hypertension','DM',

'cvd','cancer','hei2015_total_score','CKD_EPI_Scr_2009',"Year"),

, xlsx="C:\\Users\\Livek\\Desktop\\Table 03-9.xlsx")

#混合物分析###====

#WQSWQS分析----

str(d2)

names(d2)

name <- names(d2)[31:39]

# Factor(dx)

#定义数据以及锁定数据的格式（因子）

r1 <- gwqs(PhenoAgeAccel ~ wqs +age+sex+ethnicity+poverty3+BMI+marital3+home2+education+pa3+smoke+Hypertension+ DM+ cvd+ cancer+hei2015_total_score+Year+CKD_EPI_Scr_2009,mix_name = name, data = d2, q = 4,validation = 0.6,b = 1000, b1_pos = TRUE, b_constr = FALSE, seed =1003)

rn <- gwqs(PhenoAgeAccel ~ wqs +age+sex+ethnicity+poverty3+BMI+marital3+home2+education+pa3+smoke+Hypertension+ DM+ cvd+ cancer+hei2015_total_score+Year+CKD_EPI_Scr_2009,mix_name = name, data = d2, q = 4,validation = 0.6,b = 1000, b1_pos = FALSE, b_constr = FALSE, seed =1003)

#重头戏，WQS回归代码，q = 4表示采用4分位数化的自变量拟合模型；validation = 0.6，按照40%训练集和60%验证集来划分数据集；b = 1000，执行1000次bootstrap过程；b1_pos = TRUE, b1_constr = FALSE, β1效应方向限制为正（也可设置为负b1_pos = FALSE, b1_constr = TRUE）；family = "binomial"，拟合逻辑回归；seed = 1003，为了结果的可重复性，设置随机数种子（可以自行设置，同个种子出来的结果相同）。

summary(r1)

summary(rn)

#得出结果数据

gwqs_barplot(r1)

gwqs_barplot(rn)

#绘制条形图

gwqs_scatterplot(r1)

gwqs_fitted_vs_resid(r1)

#绘制散点图

ptbp<-rn$final_weights

#赋值

round(ptbp$mean_weight,4)

#四舍五入到四位小数并进行显示

gwqs_summary_tab(rn)

ptbp

#绘制表格

summary(r1$fit)

计算OR并赋值

conf_intervals <- confint(r1$fit, level = 0.95)

#WQS结果计算炎症及氧化中介效应（无阳性发现）====

library(mediation)

#"Alkaline"

#"bilirubin" "iron" "GGT"

# "SII"

#1

d2$dwqs <- d2$lnPBB153 * 0.242729328 +

d2$lnPBDE153 * 0.199349745 +

d2$lnPBDE28 * 0.198975723 +

d2$lnPBDE85 * 0.165685622 +

d2$lnPBDE209 * 0.076692233 +

d2$lnPBDE154 * 0.057348285 +

d2$lnPBDE99 * 0.034922331 +

d2$lnPBDE100 * 0.022347037 +

d2$lnPBDE47 * 0.001949698

med.fit<- lm(Alkaline ~ dwqs +age+sex+ethnicity+poverty3+BMI+marital3+home2+education+pa3+smoke+Hypertension+ DM+ cvd+ cancer+hei2015_total_score+Year+CKD_EPI_Scr_2009, data = d2)

out.fit<- lm(PhenoAgeAccel ~ Alkaline+dwqs+age+sex+ethnicity+poverty3+BMI+marital3+home2+education+pa3+smoke+Hypertension+ DM+ cvd+ cancer+hei2015_total_score+Year+CKD_EPI_Scr_2009, data = d2)

med.out<- mediate(med.fit, out.fit, treat = "dwqs", mediator ="Alkaline",

robustSE = TRUE, sims =100)###treat填自变量，mediator填中介变量

summary(med.out)

plot(med.out)

med.fit<- lm(bilirubin ~ dwqs +age+sex+ethnicity+poverty3+BMI+marital3+home2+education+pa3+smoke+Hypertension+ DM+ cvd+ cancer+hei2015_total_score+Year+CKD_EPI_Scr_2009, data = d2)

out.fit<- lm(PhenoAgeAccel ~ bilirubin+dwqs+age+sex+ethnicity+poverty3+BMI+marital3+home2+education+pa3+smoke+Hypertension+ DM+ cvd+ cancer+hei2015_total_score+Year+CKD_EPI_Scr_2009, data = d2)

med.out<- mediate(med.fit, out.fit, treat = "dwqs", mediator ="bilirubin",

robustSE = TRUE, sims =100)###treat填自变量，mediator填中介变量

summary(med.out)

plot(med.out)

med.fit<- lm(iron ~ dwqs +age+sex+ethnicity+poverty3+BMI+marital3+home2+education+pa3+smoke+Hypertension+ DM+ cvd+ cancer+hei2015_total_score+Year+CKD_EPI_Scr_2009, data = d2)

out.fit<- lm(PhenoAgeAccel ~ iron+dwqs+age+sex+ethnicity+poverty3+BMI+marital3+home2+education+pa3+smoke+Hypertension+ DM+ cvd+ cancer+hei2015_total_score+Year+CKD_EPI_Scr_2009, data = d2)

med.out<- mediate(med.fit, out.fit, treat = "dwqs", mediator ="iron",

robustSE = TRUE, sims =100)###treat填自变量，mediator填中介变量

summary(med.out)

plot(med.out)

med.fit<- lm(SII ~ dwqs +age+sex+ethnicity+poverty3+BMI+marital3+home2+education+pa3+smoke+Hypertension+ DM+ cvd+ cancer+hei2015_total_score+Year+CKD_EPI_Scr_2009, data = d2)

out.fit<- lm(PhenoAgeAccel ~ SII+dwqs+age+sex+ethnicity+poverty3+BMI+marital3+home2+education+pa3+smoke+Hypertension+ DM+ cvd+ cancer+hei2015_total_score+Year+CKD_EPI_Scr_2009, data = d2)

med.out<- mediate(med.fit, out.fit, treat = "dwqs", mediator ="SII",

robustSE = TRUE, sims =100)###treat填自变量，mediator填中介变量

summary(med.out)

plot(med.out)

med.fit<- lm(GGT ~ dwqs +age+sex+ethnicity+poverty3+BMI+marital3+home2+education+pa3+smoke+Hypertension+ DM+ cvd+ cancer+hei2015_total_score+Year+CKD_EPI_Scr_2009, data = d2)

out.fit<- lm(PhenoAgeAccel ~ GGT+dwqs+age+sex+ethnicity+poverty3+BMI+marital3+home2+education+pa3+smoke+Hypertension+ DM+ cvd+ cancer+hei2015_total_score+Year+CKD_EPI_Scr_2009, data = d2)

med.out<- mediate(med.fit, out.fit, treat = "dwqs", mediator ="GGT",

robustSE = TRUE, sims =100)###treat填自变量，mediator填中介变量

summary(med.out)

plot(med.out)

med.fit<- lm(BMI ~ dwqs +age+sex+ethnicity+poverty3+marital3+home2+education+pa3+smoke+Hypertension+ DM+ cvd+ cancer+hei2015_total_score+Year+CKD_EPI_Scr_2009, data = d2)

out.fit<- lm(PhenoAgeAccel ~ BMI+dwqs+age+sex+ethnicity+poverty3+marital3+home2+education+pa3+smoke+Hypertension+ DM+ cvd+ cancer+hei2015_total_score+Year+CKD_EPI_Scr_2009, data = d2)

med.out<- mediate(med.fit, out.fit, treat = "dwqs", mediator ="BMI",

robustSE = TRUE, sims =100)###treat填自变量，mediator填中介变量

summary(med.out)

plot(med.out)

#BKMBKMR分析----

d2 <- drop_col(d2,"age2","age3")

names(d2)

cor.matrix <- cor(d2[,29:37], method = "spearman") #计算spearman相关系数#,

corrplot.mixed(cor.matrix, number.cex = 0.8) #绘制热力图#

mixture <- data.matrix(d2[,29:37]) #暴露变量（第44列至46列）

covariates <- data.matrix(d2[,2:19]) #协变量（第17列至19列）

y1 <- d2$PhenoAgeAccel

# 设置一个非常小的随机数范围

epsilon <- 1e-10

# 生成一个与 mixture 数据框相同大小的矩阵，填充随机数

random_noise <- matrix(runif(n = nrow(mixture) * ncol(mixture), min = -epsilon, max = epsilon),

nrow = nrow(mixture), ncol = ncol(mixture))

# 在 mixture 的每个元素上加上一个小的随机数

mixture_adjusted <- mixture + random_noise

# 将调整后的数据替换到原始数据集

d2[,29:37] <- mixture_adjusted

sex=d2$sex

knots50 <- fields::cover.design(mixture_adjusted, nd = 50)$design

set.seed(100) #设置种子数（随便一个数），方便以后重复结果#

fity1 <- kmbayes(y = y1, Z =covariates , X = mixture_adjusted, iter = 10000, est.h = TRUE, verbose = FALSE, varsel = TRUE,knots = knots50)

# 男性效应

fit_male <- kmbayes(y = y1[sex == "Male"], Z = mixture_adjusted[sex == "Male", ], X = covariates[sex == "Male", ],iter = 10000, est.h = TRUE, verbose = FALSE, varsel = TRUE,knots = knots50)

# 女性效应

fit_female <- kmbayes(y = y1[sex == "Female"], Z = mixture_adjusted[sex == "Female", ], X = covariates[sex == "Female", ],iter = 10000, est.h = TRUE, verbose = FALSE, varsel = TRUE,knots = knots50)

#绘图，分别绘制参数β，误差方差以及变量之间相关系数的跟踪图。

TracePlot(fit = fity1, par = "beta")

TracePlot(fit = fity1, par = "sigsq.eps")

TracePlot(fit = fity1, par = "r", comp = 1)

#提取参数后验变量的重要性指数。1. 暴露变量的后验包含概率(PIP)PIP表示对结局影响的相对重要程度，越高表示对结局越重要

epr1 <- ExtractPIPs(fity1)

ExtractPIPs(fity1)

#2. 单变量截面 及 可视化 。

pred.resp.univar1 <- PredictorResponseUnivar(fit = fity1,q.fixed=0.5)#数据预测计算#

#固定其余13种变量水平固定在中位数时，单一变量与结局的暴露-反应截面，主要查看暴露与结局间的非线性关联

ggplot(pred.resp.univar1, aes(z, est, ymin = est - 1.96*se, ymax = est + 1.96*se)) +

geom_smooth(stat = "identity") + facet_wrap(~ variable) + ylab("h(z)") #可视化

#3. 混合物总体效应

risks.overall1 <- OverallRiskSummaries(fit = fity1, qs = seq(0.25, 0.75, by = 0.05), q.fixed = 0.5) #q.fixed：比较参考的水平#

male <- OverallRiskSummaries(fit = fit_male, qs = seq(0.25, 0.75, by = 0.05), q.fixed = 0.5) #q.fixed：比较参考的水平#

female <- OverallRiskSummaries(fit = fit_female, qs = seq(0.25, 0.75, by = 0.05), q.fixed = 0.5) #q.fixed：比较参考的水平#

risks.overall1$Group <- "Total"

male$Group <- "Male"

female$Group <- "Female"

summary_combined <- bind_rows(risks.overall1 , male, female)

mixr1 <- ggplot(risks.overall1, aes(quantile, est, ymin = est - 1.96*sd, ymax = est + 1.96*sd)) + geom_pointrange()+ geom_hline(yintercept = 0, lty = 2, col = "red")

mixr2 <- ggplot(summary_combined, aes(quantile, est, ymin = est - 1.96*sd, ymax = est + 1.96*sd)) + geom_pointrange()+ geom_hline(yintercept = 0, lty = 2, col = "red")

summary_combined$Group <- factor(summary_combined$Group, levels = c("Total", "Male", "Female"))

# Create the plot with different colors for Total, Male, and Female groups

mixr2 <- ggplot(summary_combined, aes(x = quantile, y = est, color = Group)) +

geom_pointrange(aes(ymin = est - 1.96 * sd, ymax = est + 1.96 * sd),

position = position_dodge(width = 0.1)) +

geom_hline(yintercept = 0, linetype = "dashed", color = "red") +

theme_minimal() +

labs(

x = "Quantile of Exposure",

y = "Estimated Effect",

title = "BKMR: Overall and Stratified Effects by Sex",

color = "Group"

) +

scale_color_manual(values = c("Total" = "black", "Male" = "red", "Female" = "blue"))

# Print the plot

print(mixr2)

#4. 单变量效应

risks.singvar1 <- SingVarRiskSummaries(fit = fity1, y = y1, Z = mixture, X = covariates, qs.diff = c(0.25, 0.75), q.fixed = c(0.25, 0.50, 0.75)) #qs.diff：比较不同百分位数的差值#

singler1 <- ggplot(risks.singvar1, aes(variable, est, ymin = est - 1.96*sd, ymax = est + 1.96*sd, col = q.fixed)) + geom_pointrange(position = position_dodge(width = 0.75)) + geom_hline(yintercept = 0, lty = 2, col = "red")

#5. 交互作用

pred.resp.bivar1 <- PredictorResponseBivar(fit = fity1, min.plot.dist = 1)

pred.resp.bivar.levels1 <- PredictorResponseBivarLevels(pred.resp.df = pred.resp.bivar1,Z = mixture, qs = c(0.1, 0.5, 0.9))

interr1 <- ggplot(pred.resp.bivar.levels1, aes(z1, est)) +

geom_smooth(aes(col = quantile), stat = "identity") +

facet_grid(variable2 ~ variable1) +

ggtitle("h(expos1 | quantiles of expos2)") +

xlab("expos1")

#BKMR按性别划分====

# 男性效应

fit_male <- kmbayes(Y = Y[gender == "Male"], Z = exposure_vars[gender == "Male", ], iter = 10000, family = "gaussian")

male_effect <- OverallRiskSummaries(fit = fit_male)

# 女性效应

fit_female <- kmbayes(Y = Y[gender == "Female"], Z = exposure_vars[gender == "Female", ], iter = 10000, family = "gaussian")

female_effect <- OverallRiskSummaries(fit = fit_female)

#中介分析(氧化及炎症)====

#Table S1(去除慢病)====

#Table S2(脂肪调整浓度)====

#Table S3（根据是否可以检测到2分类）====
